# Mucosal Remodeling in Chronic Rhinosinusitis with Nasal Polyps: The Role of Innate Lymphoid Cells and Reprogramming Under IL-4Rα Blockade

**DOI:** 10.3390/ijms27041992

**Published:** 2026-02-19

**Authors:** Giovanna Lucia Piazzetta, Nadia Lobello, Silvia Di Agostino, Isabella Coscarella, Corrado Pelaia, Anna Di Vito, Jessica Bria, Andrea Filardo, Annamaria Aloisio, Chiara Lupia, Nicola Lombardo, Emanuela Chiarella

**Affiliations:** 1 Otolaryngology Head and Neck Surgery, Department of Medical and Surgical Sciences, University “Magna Græcia”, 88100 Catanzaro, Italy; giovannapiazzetta@hotmail.it (G.L.P.); nadialobello@gmail.com (N.L.); 2 Department of Health Sciences, University “Magna Graecia”, 88100 Catanzaro, Italy; sdiagostino@unicz.it; 3 Department of Experimental and Clinical Medicine, University “Magna Graecia”, 88100 Catanzaro, Italy; isabella.coscarella@studenti.unicz.it (I.C.); divito@unicz.it (A.D.V.); 4 Department of Medical and Surgical Sciences, University “Magna Graecia”, 88100 Catanzaro, Italy; pelaia.corrado@unicz.it (C.P.); jessica.bria@studenti.unicz.it (J.B.); filardo@unicz.it (A.F.); aloisio@unicz.it (A.A.); chiara.lupia@hotmail.it (C.L.); 5 Department of Pharmacy, Health and Nutrition Sciences, University of Calabria, 87100 Cosenza, Italy; nicola.lombardo@unical.it

**Keywords:** innate lymphoid cells (ILCs), nasal mucosa, nasal polyps, IL-4R, dupilumab, type 2 inflammation, alarmins (IL-25, IL-33, TSLP), targeted therapy

## Abstract

The nasal mucosa functions as a highly specialized barrier that integrates epithelial, stromal, neuronal, and immune signals to maintain homeostasis and mount rapid responses to environmental challenges. Among its resident immune populations, innate lymphoid cells—particularly type 2 ILCs (ILC2s)—play a pivotal role in orchestrating type 2 inflammation driven by epithelial-derived alarmins such as IL-25, IL-33, and TSLP. Upon activation, ILC2s release IL-5 and IL-13, promoting eosinophilic inflammation, goblet cell hyperplasia, mucus hypersecretion, and tissue remodeling, all central features of chronic rhinosinusitis with nasal polyps (CRSwNP) and severe allergic rhinitis. Recent advances have revealed substantial ILC plasticity, the presence of nasal-resident ILC progenitors, and the influence of metabolic and neuroimmune cues in shaping ILC activation and persistence. Dupilumab, a monoclonal antibody targeting IL-4Rα, has emerged as a highly effective therapy, providing unique mechanistic insight into the epithelial–ILC axis. By blocking IL-4/IL-13 signaling, dupilumab dampens ILC2 effector functions, reduces IL-5/IL-13 output, restores epithelial barrier integrity, interrupts alarmin-driven amplification loops, and rebalances innate and adaptive immune networks. Clinical and translational studies indicate that baseline ILC2 phenotypes—particularly inflammatory ILC2 subsets—may predict treatment responsiveness, positioning ILC profiling as a promising biomarker strategy. This review synthesizes current knowledge of ILC classification, plasticity, progenitor biology, and epithelial–ILC communication in the nasal mucosa, while integrating emerging evidence on dupilumab-mediated immunomodulation. Collectively, these insights highlight ILCs as central drivers of type 2 inflammation and key targets for precision immunomodulation, offering a framework for personalized treatment approaches in CRSwNP and allergic rhinitis.

## 1. Introduction

The nasal cavity is the uppermost segment of the respiratory tract, acting as a sentinel site for airborne particles, allergens, and pathogens. It functions as a primary interface between the host and the external environment. Its mucosa is a specialized environment composed of epithelial, stromal, vascular, and immune components that cooperate to maintain tissue integrity and homeostasis [1,2]. Historically, mucosal immunity research has centered on adaptive lymphocytes, particularly T helper (Th) cells [3,4,5,6]. The identification of innate lymphoid cells (ILCs) has substantially reshaped this paradigm, revealing a rapid, tissue-resident immune compartment that operates upstream of adaptive immunity at barrier sites [7,8].

ILCs are lymphoid cells lacking antigen-specific receptors but capable of rapid cytokine production, mirroring Th subsets in function [9,10]. Within the nasal mucosa, ILCs localize beneath the epithelium, positioning them as key regulators of early immune activation and tissue responses [11,12]. ILC2s dominate this niche and orchestrate type 2 inflammation that is central to allergic rhinitis and CRSwNP [13,14]. By secreting IL-5 and IL-13, ILC2s promote eosinophilic infiltration, goblet cell hyperplasia, and mucus overproduction [15,16]. Beyond cytokine secretion, ILC2s engage in dynamic crosstalk with epithelial cells, fibroblasts, neurons, and eosinophils, forming integrated cellular networks that sustain chronic inflammation and drive tissue remodeling over time [17,18,19]. These observations position ILC biology at the interface between immune dysregulation and the architectural changes that underlie polyp growth and disease chronicity in CRSwNP [17,18,19].

The introduction of dupilumab, an IL-4Rα-blocking antibody, has improved clinical outcomes in patients with CRSwNP, offering novel research scenarios for the study of innate immune regulation. Dupilumab, a monoclonal antibody targeting the IL-4 receptor alpha (IL-4Rα) subunit shared by IL-4 and IL-13 receptors, has markedly improved clinical outcomes in patients with severe CRSwNP and has provided a unique opportunity to interrogate the role of type 2 immune pathways in mucosal disease [19,20,21,22,23]. Beyond its therapeutic efficacy, dupilumab treatment has revealed how modulation of epithelial–ILC interactions can recalibrate local immune responses and promote the restoration of tissue homeostasis [24]. Overall, dupilumab has demonstrated a favorable safety profile in both clinical trials and real-world studies [25,26,27]; however, rare cases of lymphoid neoplasms have been reported in patients treated for atopic dermatitis, based on isolated case reports, without evidence of a causal relationship [28,29,30,31]. Studies investigating ILC modulation under dupilumab therapy have highlighted how restoring epithelial–ILC balance recalibrates nasal immunity and supports tissue homeostasis.

In this context, mucosal remodeling is a central pathological hallmark of CRSwNP, encompassing epithelial barrier disruption, goblet cell hyperplasia, stromal edema, extracellular matrix reorganization, and polyp formation [32]. Disruption of this finely tuned mucosal system drives persistent inflammation and structural changes, with ILC2-derived cytokines—particularly IL-5 and IL-13—acting as key mediators by modulating epithelial differentiation, fibroblast activation, and eosinophil–stromal interactions [33,34]. Accordingly, ILC biology is not only central to inflammatory signaling but also to the mechanisms sustaining chronic tissue remodeling in the nasal mucosa [35].

This review aims to integrate recent insights from mucosal immunology and clinical studies to elucidate how ILC plasticity, epithelial–immune interactions, and dupilumab-mediated modulation converge to redefine nasal immune homeostasis. By framing ILCs within a remodeling-centered paradigm, we aim to provide a biologically grounded perspective on disease mechanisms and emerging therapeutic strategies.

## 2. Innate Lymphoid Cells and Progenitors in the Nasal Mucosa: Classification, Plasticity, and Functional Roles

### 2.1. ILC Subsets, Development, and Local Progenitors in the Nasal Mucosa

Innate lymphoid cells (ILCs) represent a heterogeneous family of lymphocyte-like innate immune cells that, despite lacking rearranged antigen receptors, exert key functions in early immune defense, tissue surveillance, and barrier protection [36,37]. Unlike T and B cells, they are equipped with pre-configured sensory programs enabling the rapid detection of cytokines, epithelial alarmins, and neuropeptides. This allows them to respond almost immediately to allergens, pathogens, or mechanical damage in mucosal tissues, including the nasal cavity, where they serve as first-line sentinels [35,38,39,40,41].

#### 2.1.1. ILC Subsets in the Nasal Mucosa

In the nasal mucosa, ILCs are enriched beneath the epithelium and within the lamina propria, a location that affords them direct access to environmental signals. They are conventionally divided into three major functional groups—ILC1s, ILC2s, and ILC3s—mirroring the effector profiles of Th1, Th2, and Th17 cells, respectively [27]. ILC1s, characterized by T-bet expression, produce interferon-γ and contribute to antiviral defense and immunity against intracellular pathogens [42,43]. ILC2s depend on GATA-3 and generate IL-5, IL-9, and IL-13 in response to epithelial alarmins such as IL-25, IL-33, and TSLP, positioning them as primary drivers of allergic inflammation, mucus hypersecretion, and epithelial remodeling [44,45]. ILC3s require RORγt for their development and secrete IL-17A, IL-17F, and IL-22, thereby promoting epithelial repair, barrier stability, and protection against extracellular bacteria and fungi [44,46]. Although historically grouped within the type 1 ILC family, natural killer (NK) cells represent a distinct lineage specialized in cytotoxicity, relying on perforin- and granzyme-mediated killing and guided by transcription factors such as Eomes and T-bet [43,47]. Their presence in the upper airway complements the canonical ILC subsets by providing additional antiviral, antibacterial, and antitumor surveillance.

#### 2.1.2. Developmental Pathways from Bone Marrow to Mucosal Surfaces

**Figure 1:**
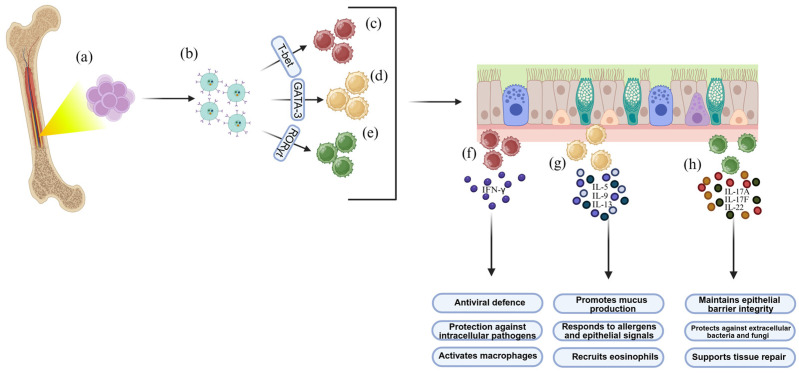
Ontogeny and Functional Specialization of Innate Lymphoid Cells in the Nasal Mucosa. ILCs are generated from a common lymphoid progenitor in the bone marrow (**a**), from which ILCps develop (**b**). These then differentiate into ILC1 (**c**), ILC2 (**d**), and ILC3 (**e**) under the influence of transcription factors such as T-bet, GATA3, and RORγt, respectively. Within the nasal mucosa, these cells perform distinct functions: ILC1, through the production of IFN-γ, contribute to antiviral defense, protection against intracellular pathogens, and macrophage activation (**f**); ILC2, through the secretion of IL-5, IL-9, and IL-13, promote mucus production, respond to allergens and epithelial signals, and recruit eosinophils (**g**); ILC3, through the secretion of IL-17A, IL-17F, and IL-22, help maintain epithelial barrier integrity, protect against extracellular bacteria and fungi, and support tissue repair (**h**). Although IL-4 is not a primary cytokine produced by ILC2s, it contributes to type 2 inflammation by amplifying Th2 polarization and enhancing IL-13-dependent epithelial and stromal remodeling, thereby reinforcing mucosal pathology in CRSwNP.Created in BioRender. Chiarella, E. (2026) https://BioRender.com/x3d7b2m (accessed on 11 February 2026).

ILCs originate from common lymphoid progenitors (CLPs) in the bone marrow and progress through an intermediate stage known as the ILC progenitor (ILCp), defined by IL-7Rα, α4β7, and transcription factors such as PLZF, TOX, and ID2 (as shown in Figure 1a,b). Although already committed to the ILC lineage, these progenitors retain the capacity to differentiate into ILC1, ILC2, or ILC3 depending on local tissue cues [44,46,47,48,49]. The early innate lymphoid progenitor (EILP), marked by α4β7, Tcf7, Nfil3, Tox, Id2, Rora, and Gata3, gives rise to the common helper innate lymphoid progenitor (CHILP), which in turn generates the helper ILC subsets and lymphoid tissue inducer (LTi) cells [41,50,51,52].

From CHILP, two main pathways diverge: one leading to LTi progenitors, which express increasing levels of RORγt and participate in lymphoid tissue formation, and another giving rise to innate lymphoid cell progenitors (ILCPs), characterized by α4β7^+^ Thy1^+^ CD127^+^ PD-1^+^ and transcription factors such as PLZF (Zbtb16), Gata3, Id2, Tcf7, Tox, Runx, and Rora [53,54,55,56,57]. Depending on the cytokine milieu and local tissue cues, ILCPs then differentiate into ILC1s, ILC2s, or ILC3s (as shown in Figure 1c–e), with recent methodological advances—including efficient lentiviral gene-delivery systems—have further enabled detailed investigation of these developmental programs [41,58,59].

IL-7 is indispensable for the development of all helper ILCs, especially ILC2s and ILC3s, whereas IL-15 primarily supports NK-cell and certain ILC1 subset maturation [60,61,62,63,64,65]. Terminal differentiation is marked by the acquisition of lineage-defining transcription factors: T-bet for ILC1s, GATA-3 and RORα for ILC2s, and sustained RORγt expression for ILC3s [66,67,68,69,70,71,72]. These transcriptional programs tightly dictate the production of IFN-γ by ILC1s, IL-5/IL-9/IL-13 by ILC2s, and IL-17A/IL-17F/IL-22 by ILC3s (as shown in Figure 1f–h) [10,73].

After maturation, ILCs migrate from the bone marrow to peripheral tissues, including the upper airway, where epithelial-derived IL-7, IL-25, IL-33, TSLP, and retinoic acid refine their phenotype and functional specialization.

#### 2.1.3. Tissue Residency, Local Progenitors, and Homeostatic Roles in the Nasal Mucosa

Increasing evidence indicates that ILC progenitors are not restricted to the bone marrow. ILCPs have been identified in several peripheral tissues, including the nasal mucosa, where they respond to epithelial and stromal mediators—such as IL-7, IL-1β, and retinoic acid—to generate mature ILC subsets in situ [74,75,76]. This local differentiation capacity suggests that the nasal mucosa maintains partial autonomy in replenishing and shaping its innate immune compartment, enabling rapid adaptations to environmental challenges without relying solely on continual recruitment from central hematopoietic organs [65,76,77,78,79]. Under homeostatic conditions, ILC2s constitute the predominant subset in the nasal mucosa [80]. Their basal secretion of IL-5 and IL-13, along with amphiregulin release, supports epithelial integrity and promotes steady-state repair processes. ILC3s contribute to structural maintenance through IL-22-mediated enhancement of tight junctions, mucin synthesis, and antimicrobial peptide production. Although less abundant, ILC1s and NK cells remain crucial for early antiviral defense via IFN-γ production and cytotoxic activity [81,82,83].

Together, these populations form an integrated surveillance system that interprets epithelial, microbial, and neuronal cues to maintain epithelial barrier stability and coordinate with adaptive immunity when necessary. The presence of local ILCPs ensures that this network remains dynamic, resilient, and capable of adapting to both acute insults and ongoing tissue maintenance.

### 2.2. Plasticity and Functional Reprogramming of Nasal ILCs

ILCs within the nasal mucosa exhibit a high degree of phenotypic plasticity, allowing them to adopt new functional states or even undergo trans-differentiation in response to cytokine gradients, microbial products, or signals of tissue injury. This adaptability is essential for quickly responding to the fluctuating environmental demands of the upper airway; however, dysregulation of these reprogramming processes may promote chronic inflammation and disrupt mucosal homeostasis [84,85,86].

A well-characterized example is the conversion of ILC2s into ILC1-like cells under the influence of IL-12 and IL-18 [86,87]. These cytokines induce T-bet expression and enhance IFN-γ production, conferring type 1—like features on formerly type 2—polarized cells [42]. This shift frequently occurs during viral infections or under persistent epithelial stress, representing an adaptive mechanism for reinforcing antiviral immunity. Conversely, alarmins such as IL-33 and TSLP preserve GATA-3 expression and stabilize the classical ILC2 phenotype, counteracting the drift toward a type 1 profile [42,86,88].

ILC3s also display considerable plasticity and can acquire ILC1-like characteristics under inflammatory conditions rich in IL-1β, IL-12, or IL-23. Downregulation of RORγt accompanies their transition toward IFN-γ production, leading to the loss of epithelial-supportive functions and the promotion of pro-inflammatory responses that may impair tissue repair. Such changes are often implicated in the perpetuation of epithelial barrier dysfunction in chronic upper airway inflammatory diseases [42,89,90,91,92].

Single-cell RNA sequencing and high-dimensional cytometry uncovered transitional populations, including GATA-3^+^T-bet^+^ intermediate ILC2/ILC1 states. These hybrid populations often expand in severe or steroid-refractory airway inflammation, where the microenvironment imposes mixed effector pressures. Their ability to simultaneously produce type 1 and type 2 cytokines may exacerbate inflammation and contribute to treatment resistance [91,93,94,95].

At the mechanistic level, ILC plasticity relies on epigenetic accessibility at key transcription factor loci, enabling rapid and potentially reversible modulation of effector programs. In the nasal mucosa—a tissue continuously exposed to allergens, microbes, pollutants, and epithelial alarmins—this epigenetic flexibility provides a powerful means of adaptation while also creating a vulnerability in which persistent inflammatory signals can drive maladaptive reprogramming and chronic disease [96,97].

### 2.3. Metabolic and Neuro-Immune Regulation of ILCs in the Nasal Mucosa

ILC development and function in the nasal mucosa are deeply influenced by metabolic constraints and by intricate neuro-immune circuits that integrate epithelial, stromal, and sensory inputs. In inflamed or obstructed nasal tissues, edema and reduced airflow often create hypoxic niches that stabilize HIF-1α and HIF-2α. These transcription factors reprogram cellular metabolism toward glycolysis, a shift that supports rapid ILC2 activation and sustains high-level IL-5 and IL-13 production even under metabolic stress [98,99,100,101,102,103]. This facilitates the amplification of type 2 inflammation. On the other hand, metabolites such as retinoic acid and short-chain fatty acids can attenuate ILC activity by promoting regulatory pathways and enhancing tissue repair, acting as counterbalances to excessive inflammatory responses [104].

Furthermore, neuronal inputs modulate ILC behavior. Sensory fibers innervating the nasal mucosa release neuropeptides—including neuromedin U (NMU), vasoactive intestinal peptide (VIP), and calcitonin gene-related peptide (CGRP)—that directly influence ILC activation states. NMU engages NMUR1 on ILC2s and induces potent activation accompanied by robust type 2 cytokine production [105,106,107]. VIP exerts context-dependent effects, at times promoting ILC2-mediated inflammation and at others supporting epithelial repair, depending on the prevailing cytokine environment [108,109]. CGRP, primarily released by trigeminal sensory neurons, displays bidirectional activity: it can enhance or suppress ILC functions based on concomitant inflammatory cues [110,111].

Lipid mediators provide yet another regulatory layer. Prostaglandin D_2_ (PGD_2_), produced by mast cells and epithelial cells, binds CRTH2 on ILC2s to enhance their recruitment and augment IL-5 and IL-13 secretion. In contrast, specialized pro-resolving mediators such as resolvins limit ILC2 activation and promote the resolution of inflammation, highlighting the delicate equilibrium between pro-inflammatory and pro-resolving lipid pathways [112,113,114,115].

Collectively, the integration of metabolic, neuro-immune, and lipid-derived signals shapes ILC behavior, survival, and effector functions within the nasal microenvironment. This multilayered regulatory network ultimately determines whether ILCs promote epithelial protection and homeostasis or drive persistent type 2 inflammation, offering insights into therapeutic strategies aimed at modulating ILC activity in chronic nasal inflammatory diseases.

## 3. Epithelial–ILC Crosstalk in the Nasal Mucosa

The nasal epithelium acts not only as a mechanical barrier but also as an active immunological sensor that continuously evaluates inhaled environmental stimuli and shapes local immune responses [116,117]. When exposed to allergens, pathogens, pollutants, or mechanical stress, epithelial cells rapidly release alarmins—including IL-33, IL-25, and TSLP—which represent the primary upstream signals activating ILC2s [118]. IL-33 engages the ST2 receptor on ILC2s, initiating MyD88-dependent NF-κB and MAPK pathways, whereas IL-25 and TSLP signal through IL-17RB and IL-7Rα receptor complexes, respectively. Together, these pathways induce rapid secretion of IL-5 and IL-13, cytokines that drive eosinophil recruitment, goblet cell hyperplasia, mucus hyperproduction, and tissue remodeling [119,120,121,122]. This establishes a self-reinforcing circuit that supports type 2 inflammation and contributes to diseases such as CRSwNP and severe allergic rhinitis [35,116,123,124].

Although IL-5 and IL-13 are the dominant effector cytokines produced by ILC2s, IL-4 plays a complementary and amplifying role in type 2 inflammation and nasal mucosal remodeling. Unlike IL-5 and IL-13, IL-4 is not primarily produced by ILC2s but is mainly derived from Th2 cells, basophils, and mast cells. IL-4 acts upstream in the type 2 cascade by promoting Th2 differentiation, IgE class switching, and sustained responsiveness of epithelial and stromal cells to IL-13. In the nasal mucosa, IL-4 enhances IL-13-dependent epithelial remodeling and indirectly contributes to mucus hypersecretion and barrier dysfunction by reinforcing type 2 immune polarization [118,125].

Beyond classical cytokine–receptor communication, epithelial–ILC interactions are influenced by neuroimmune signals and lipid mediators, which add complexity to the regulation of ILC activation. Neuropeptides, including NMU, VIP, and CGRP, can either amplify or restrain ILC2 activity depending on the microenvironmental context, thereby linking neuronal sensing to immune modulation. Lipid mediators such as PGD_2_ further enhance ILC2 chemotaxis and cytokine release through CRTH2 engagement, integrating environmental and neural inputs to fine-tune the magnitude and spatial distribution of type 2 responses [107,114,126,127].

ILCs also influence the adaptive immune system through multiple forms of crosstalk. IL-13 produced by activated ILC2s conditions dendritic cells to promote Th2 polarization, while IL-5 supports eosinophil maturation and survival, reinforcing a local inflammatory circuit [128,129]. In this context, IL-4 further sustains adaptive type 2 amplification by stabilizing Th2 identity and supporting IgE-mediated effector pathways, thereby indirectly contributing to chronic inflammation and tissue remodeling [130]. Moreover, ILCs directly interact with epithelial cells and fibroblasts to regulate epithelial barrier integrity and tissue remodeling, while regulatory mechanisms involving Tregs or IL-10-producing ILC subsets help limit excessive inflammation [83,88,131,132]. In chronic nasal diseases, however, these regulatory mechanisms often fail or become overwhelmed, contributing to persistent inflammation and polyp formation [118].

Recent findings suggest that nasal-resident ILC progenitors (ILCp) also participate in the dialogue between the epithelium and the innate lymphoid compartment. ILCp can sense epithelial-derived alarmins and other local cytokines, differentiating in situ into mature ILC2s or ILC3s. This localized differentiation provides the mucosa with the capacity to rapidly adapt its innate immune repertoire to environmental challenges, supporting both acute defense and chronic inflammatory remodeling [18,85,118].

Metabolic and microenvironmental cues further regulate epithelial–ILC communication. Hypoxia—commonly present in obstructed or inflamed nasal passages—induces HIF protein stabilization in ILC2s, favoring glycolysis and enhancing the effector functions. Nutrient availability, metabolite accumulation, and oxidative stress all influence cytokine production, survival, and plasticity. Importantly, plasticity enables ILC2s exposed to IL-12 or IL-18 to acquire ILC1-like features, while certain inflammatory contexts can drive ILC3-to-ILC1 transitions. These shifts help the tissue adapt to mixed inflammatory milieus but may also perpetuate chronic pathology [101,103,133,134].

**Figure 2:**
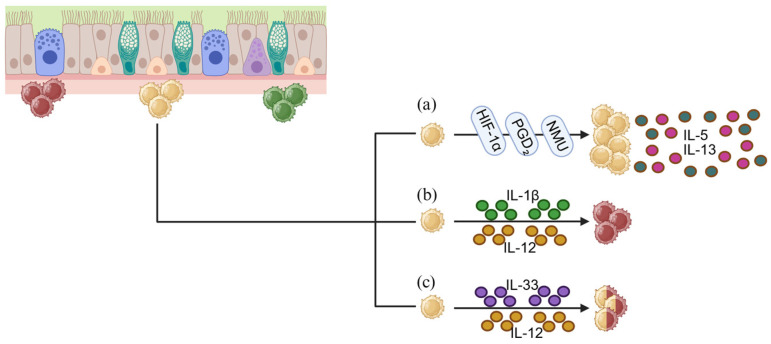
Microenvironmental Regulation of ILC2 Activation and Plasticity. The microenvironment influences the expression and plasticity of ILC2. The presence of neuropeptides such as NMU, lipid mediators like PGD_2_, and hypoxia—commonly found in obstructed or inflamed nasal passages—promotes persistent ILC2 activation, leading to robust production of IL-5 and IL-13 (**a**). Cytokines such as IL-12 and IL-1β drive the conversion of ILC2 into ILC1 (**b**), while prolonged inflammation resulting from the co-existence of IL-12 and type 2 alarmins like IL-33 promotes the formation of ILC1/ILC2 hybrid cells (**c**). Created in BioRender. Chiarella, E. (2026) https://BioRender.com/szr2gnr (accessed on 11 February 2026).

In CRSwNP and allergic rhinitis, epithelial–ILC crosstalk becomes pathologically amplified. CRSwNP is characterized by marked expansion and activation of ILC2s beneath the epithelium and within the lamina propria, driven by persistent exposure to IL-33, IL-25, TSLP, and microbial signals. These activated ILC2s produce abundant IL-5 and IL-13, sustaining eosinophilia, mucus hypersecretion, epithelial remodeling, and ultimately polyp formation (as shown in Figure 2a) [35]. Within this inflammatory milieu, IL-4 contributes to the maintenance of a permissive type 2 environment by reinforcing Th2-driven immune circuits and amplifying IL-13-dependent remodeling responses [34]. In allergic rhinitis, particularly in severe or refractory cases, increased ILC2 frequencies and heightened type 2 cytokine production contribute to sustained mucosal edema and, in some patients, polypoid transformation [13]. Thus, ILC2s act as central regulators of type 2 inflammatory pathology across a spectrum of nasal diseases.

ILC plasticity further shapes disease severity. Hybrid ILC2/ILC1 populations—co-expressing GATA3 and T-bet and capable of producing both IL-13 and IFN-γ—have been identified in refractory CRSwNP and steroid-resistant airway disease [35]. These cells arise in contexts where type 2 alarmins coexist with IL-12 or other type 1—skewing cytokines and contribute to prolonged inflammation through combined type 1/2 effector profiles [86]. Likewise, chronic inflammation can promote ILC3-to-ILC1 transitions, exacerbating epithelial damage and hindering effective mucosal repair [135].

The nasal mucosa microenvironment strongly influences these processes. IL-12 and IL-1β promote ILC2-to-ILC1 conversion, IL-23 maintains ILC3 identity, and IL-33 and IL-25 drive ILC2 expansion. Simultaneously, neuropeptides, lipid mediators, stromal remodeling, and hypoxia-driven metabolic changes sustain a milieu that favors persistent ILC activation. Nasal-resident ILCp further reinforce chronic inflammation by continuously supplying new effector ILCs, highlighting why some patients experience recurrent or persistent disease despite therapy (as shown in Figure 2b,c) [89,136].

Overall, epithelial–ILC crosstalk in the nasal mucosa forms an intricate network in which alarmin signaling, neural inputs, metabolic cues, and progenitor dynamics converge to determine whether ILCs maintain tissue homeostasis or drive chronic inflammation and remodeling. Within this framework, IL-4 functions as an essential amplifier of type 2 immune polarization rather than a primary ILC2 effector cytokine, explaining its indirect yet biologically relevant contribution to nasal mucosal remodeling. These insights have clear translational implications. Therapeutic agents targeting upstream mediators of type 2 inflammation—particularly the IL-4Rα-blocking antibody dupilumab—can interrupt the epithelial–ILC amplification loop, reduce pathogenic ILC2 activity, and restore epithelial function. By recalibrating the inflammatory microenvironment, dupilumab not only alleviates symptoms but also modulates ILC behavior, underscoring the importance of understanding epithelial–ILC communication and ILC plasticity in chronic nasal disease [21,24,137].

### 3.1. Targeted Therapy in Nasal Inflammation: Mechanism of Action of Dupilumab and Therapeutic Relevance

Biologic therapies have substantially advanced the management of chronic rhinosinusitis with nasal polyps (CRSwNP) by selectively targeting key mediators of type 2 inflammation. Among these, monoclonal antibodies directed against IL-5 or its receptor—such as mepolizumab and benralizumab—have demonstrated efficacy in reducing eosinophil differentiation, survival, and tissue infiltration [138,139]. Because IL-5 represents a major effector cytokine produced by activated ILC2s, IL-5 blockade effectively attenuates eosinophil-driven inflammation and improves clinical outcomes in patients with eosinophil-dominant CRSwNP endotypes [140]. Nevertheless, IL-5-targeted therapies primarily act downstream within the type 2 inflammatory cascade and do not directly interfere with upstream epithelial activation, alarmin release, or ILC2 priming. As a consequence, their effects on epithelial remodeling and barrier dysfunction may be indirect or incomplete. In contrast, therapies targeting IL-4Rα inhibit both IL-4- and IL-13-mediated signaling pathways, thereby exerting broader effects on epithelial cells, innate lymphoid cells, and adaptive Th2 responses [141]. This upstream mode of action provides a mechanistic framework to investigate how modulation of the epithelial–ILC axis can reshape mucosal inflammation and remodeling, which is the focus of the following sections.

#### 3.1.1. Molecular Basis of IL-4Rα Blockade

**Figure 3:**
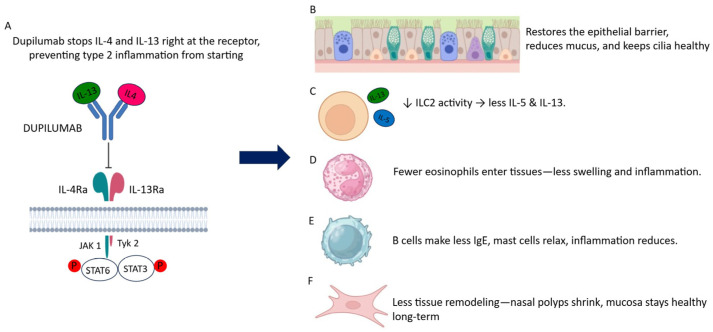
Schematic representation of the impact of IL-4Rα blockade on epithelial and innate lymphoid cell (ILC) pathways in the nasal mucosa. (**A**) In inflamed nasal mucosa, epithelial cells are exposed to IL-4 and IL-13 signaling through the IL-4Rα receptor, contributing to epithelial dysfunction and sustained type 2 inflammation. (**B**) IL-4/IL-13 signaling promotes the activation of tissue-resident type 2 innate lymphoid cells (ILC2s), characterized by GATA-3 expression and an activated effector phenotype. (**C**) Activated ILC2s produce type 2 cytokines, particularly IL-5 and IL-13, which amplify local inflammation, promote eosinophilic responses, and further impair epithelial barrier function. (**D**) Dupilumab binds to IL-4Rα, inhibiting IL-4- and IL-13-dependent signaling in epithelial cells and ILC2s. (**E**) IL-4Rα blockade also attenuates type 2—driven adaptive immune responses, including reduced activation and IgE-promoting signaling in B lymphocytes, contributing to the suppression of humoral type 2 amplification loops. (**F**) The combined effects of IL-4Rα inhibition lead to restoration of epithelial barrier integrity and rebalancing of the local inflammatory microenvironment toward tissue homeostasis. Created in BioRender. Chiarella, E. (2026) https://app.biorender.com/citation/69924767287528204fa9ff7b (accessed on 11 February 2026).

Dupilumab is a fully human IgG4 monoclonal antibody that selectively binds the interleukin-4 receptor alpha subunit (IL-4Rα), thereby preventing its heterodimerization with the common γ chain (γc) or with IL-13Rα1 [142,143,144]. Through this single molecular interaction, dupilumab inhibits signaling mediated by both IL-4 and IL-13, two cytokines that form the central hub of type 2 inflammation across epithelial, innate, and adaptive compartments (as shown in Figure 3A). By neutralizing IL-4/IL-13 signaling at this shared receptor, dupilumab effectively disrupts one of the earliest checkpoints of type 2 immune activation, making its mechanism broader and more upstream than that of cytokine-specific biologics. By blocking IL-4Rα, dupilumab interrupts the activation of JAK1-dependent pathways and abolishes downstream STAT6 phosphorylation, ultimately suppressing the transcription of genes involved in eosinophil recruitment, mucus hypersecretion, IgE class switching, epithelial remodeling, and survival programs sustaining innate lymphoid cells. This upstream blockade not only prevents downstream inflammatory cascades but also dampens epithelial stress responses, contributing to more rapid clinical improvement [142,145].

#### 3.1.2. Effects on Epithelial Cells

Epithelial cells in the nasal mucosa express IL-4Rα/IL-13Rα1 and are highly responsive to IL-4 and IL-13, which act as potent modulators of epithelial differentiation, mucus production, and epithelial barrier integrity. Under type 2 cytokine stimulation, epithelial cells undergo goblet cell metaplasia, increased mucin gene transcription, reduced ciliary function, and enhanced release of pro-inflammatory chemokines and alarmins such as IL-33, TSLP, and IL-25 [146]. Dupilumab counteracts these deregulated pathways by restoring STAT6-independent differentiation programs, re-establishing tight-junction integrity, and normalizing epithelial turnover. Furthermore, IL-4Rα inhibition helps stabilize epithelial–mesenchymal interactions, reducing mechanical cues that perpetuate chronic mucosal remodeling. As IL-4/IL-13-driven epithelial stress decreases, the production of alarmins diminishes, relieving continuous activation of tissue-resident ILC2s [21,147]. This epithelial re-equilibration is clinically reflected in reduced mucus hypersecretion, improved airflow, and restoration of olfactory epithelial function (as shown in Figure 3B). These early epithelial changes are considered among the strongest contributors to the rapid symptomatic relief observed during dupilumab therapy [148,149].

#### 3.1.3. Effects on ILC2s and Innate Immunity

ILC2s are among the most responsive cell types to IL-4 and IL-13, which reinforce GATA-3 expression, maintain effector polarization, and enhance responsiveness to IL-33, IL-25, and TSLP. Within the nasal mucosa, these cytokines foster ILC2 survival, proliferation, and sustained production of IL-5 and IL-13, creating a feed-forward loop of eosinophilic inflammation and epithelial dysfunction [34,150]. Dupilumab interferes with this loop by withdrawing essential cytokine support for activated ILC2s (as shown in Figure 3C). As IL-4Rα-dependent signals diminish, ILC2s exhibit reduced activation marker expression, downregulated CRTH2 and CD69, and a marked decline in IL-5 and IL-13 secretion [34,138]. Notably, this reduction in ILC2 effector output occurs without triggering apoptosis, indicating a functional “quiescence” rather than depletion. Transcriptomic studies indicate that IL-4Rα blockade shifts ILC2s toward a less effector-polarized state without forcing transdifferentiation, thereby attenuating their pathogenic potential while preserving baseline homeostatic functions. This rapid modulation of the innate compartment aligns with the early clinical benefits reported in CRSwNP patients treated with dupilumab [34,138].

However, it remains unclear whether this quiescent state induced by IL-4Rα blockade is sustained after treatment cessation or represents a transient suppression of effector function. Understanding this distinction is pivotal in considering whether dupilumab may confer disease-modifying effects rather than solely symptomatic control.

Emerging evidence suggests that ILC2s can acquire features of “innate immune memory,” characterized by durable changes in functional responsiveness upon repeated stimulation. In both experimental models and human studies, subsets of ILC2s have been shown to persist and respond more vigorously upon secondary challenge, with associated changes in chromatin accessibility that underlie trained or memory-like states [151].

Mechanistically, repetitive inflammatory stimulation leads to epigenetic reprogramming in ILC2s, involving alterations in chromatin landscapes (e.g., changes in accessibility at key loci such as TLR4 and other memory-associated gene motifs), implicating transcriptional regulators and chromatin modifiers in establishing persistent functional states [152]. These findings raise the possibility that IL-4Rα blockade with dupilumab might not only suppress effector cytokine production acutely but could also influence the epigenetic “inflammatory memory” of ILC2s, potentially diminishing their propensity for reactivation after allergen exposure or treatment discontinuation. Although direct evidence for long-term epigenetic reprogramming of ILC2s in the context of IL-4Rα inhibition is not yet established, the concept aligns with broader observations of innate immune memory in ILC subsets and provides a possible framework for future investigation. Thus, modulation of ILC2s by dupilumab might extend beyond transient effector suppression toward a more durable recalibration of innate immune responsiveness, with implications for disease modification and tolerance.

#### 3.1.4. Effects on Eosinophils and Granulocytes

Although IL-5 remains the principal cytokine responsible for eosinophil differentiation and survival, IL-4 and IL-13 indirectly regulate eosinophil biology by inducing epithelial and stromal production of chemokines such as eotaxins (CCL11, CCL24, CCL26). By neutralizing IL-4Rα signaling, dupilumab reduces eotaxin production, limits eosinophil recruitment, and diminishes tissue eosinophilia (as shown in Figure 3D). This attenuation of eosinophil trafficking contributes significantly to reductions in stromal edema and glandular hyperplasia. In nasal polyposis, this results in reduced epithelial edema and attenuation of eosinophil-driven tissue remodeling [21]. While peripheral eosinophil counts may transiently rise in some patients as a result of altered trafficking dynamics, tissue eosinophils consistently decline, correlating with structural and symptomatic improvement. This transient peripheral eosinophilia is generally considered a benign redistribution phenomenon rather than a marker of increased inflammation [20].

#### 3.1.5. Effects on B Cells and IgE Pathways

IL-4 is essential for immunoglobulin class switching to IgE, while IL-13 supports IgE amplification and influences stromal and epithelial IgE-dependent responses. Dupilumab inhibits these signaling pathways, leading to a progressive reduction in total and local IgE production (as shown in Figure 3E) [21]. Although IgE levels decrease more slowly than other markers of type 2 inflammation, their gradual decline reflects a sustained inhibition of IL-4-driven humoral immunity [21,145]. Although IgE normalization proceeds more slowly compared to cytokine suppression, the reduction in IgE-dependent mast-cell activation contributes to lower mediator release, reduced neurogenic inflammation, and diminished amplification of type 2 effector circuits within the nasal mucosa. This long-term modulation of IgE biology adds an additional layer of therapeutic benefit beyond the immediate suppression of type 2 cytokine activity [20].

#### 3.1.6. Effects on Stromal Cells, Fibroblasts, and Tissue Remodeling

Beyond immune regulation, IL-4 and IL-13 are key profibrotic cytokines that act on fibroblasts and structural cells to promote collagen deposition, extracellular matrix remodeling, periostin upregulation, and subepithelial fibrosis (as shown in Figure 3F) [34]. These remodeling pathways are central to polyp formation and recurrence in CRSwNP [34,153,154]. By interrupting IL-4Rα signaling in stromal cells, dupilumab attenuates fibroblast activation and matrix gene transcription, progressively reducing the structural drivers of nasal polyp growth [142]. This effect is particularly relevant for long-standing disease, in which fibrotic and stromal changes are major contributors to symptom persistence. Imaging studies and histopathology confirm that polyp regression during therapy correlates with molecular signatures of reduced type 2-driven remodeling and normalization of epithelial–stromal interactions. Over time, these modifications support a more stable and less relapse-prone mucosal architecture [155].

Clinical trials and translational studies have consistently shown that IL-4Rα blockade leads to a significant reduction in type 2 inflammatory biomarkers—including total IgE, eotaxin-3, IL-5, and IL-13—in nasal secretions, serum, and polyp tissue. These molecular changes occur early during treatment and are associated with reductions in polyp size, mucosal edema, and radiologic disease burden, indirectly reflecting improved epithelial–stromal interactions and tissue architecture [20,143].

From a histopathological perspective, direct quantitative assessments of classical remodeling features—such as basement membrane thickening, subepithelial fibrosis, or collagen deposition—before and after dupilumab therapy remain limited. However, emerging real-world and biopsy-based observations indicate that modulation of the IL-4/IL-13 axis influences stromal remodeling pathways. In particular, residual or treatment-refractory polyp tissues have been reported to display increased deposition of extracellular matrix components and enhanced expression of periostin, a matricellular protein strongly induced by IL-13 and implicated in fibrotic remodeling, suggesting that structural remodeling may evolve more slowly than inflammatory suppression and may differ according to individual response patterns [155].

The involvement of periostin in CRSwNP remodeling is biologically plausible and well supported. Elevated periostin expression in nasal polyp tissue correlates with epithelial barrier disruption, increased basement membrane thickness, goblet cell hyperplasia, and eosinophilic infiltration—hallmark features of chronic mucosal remodeling [156]. Because periostin is produced mainly by activated fibroblasts in response to IL-4 and IL-13 signaling, its modulation represents a key link between immune dysregulation and structural tissue changes.

In parallel, transforming growth factor-β (TGF-β) signaling plays a central role in regulating extracellular matrix turnover and fibrosis in CRSwNP. Histological studies have demonstrated altered spatial and cellular patterns of TGF-β expression in nasal polyps, particularly within the subepithelial stroma, contributing to abnormal collagen deposition and tissue stiffness. By inhibiting upstream IL-4/IL-13-dependent fibroblast activation, dupilumab may indirectly influence TGF-β-driven remodeling pathways, thereby promoting gradual normalization of stromal architecture over time [157].

Overall, while dupilumab clearly induces rapid immunological reprogramming and clinical improvement, robust evidence of complete reversal of established histological remodeling remains limited. Current data support a model in which IL-4Rα blockade initiates early epithelial and immune normalization, followed by slower, progressive modulation of fibrosis-related pathways, highlighting the need for longitudinal biopsy studies incorporating standardized histological scoring systems to better define the extent and durability of mucosal remodeling under biologic therapy.

#### 3.1.7. Integrated Clinical and Biological Implications

The combined effects of IL-4Rα blockade on epithelial cells, ILC2s, eosinophils, B cells, and stromal elements create a coordinated reprogramming of the nasal mucosal microenvironment. Early clinical improvements largely mirror rapid suppression of epithelial dysfunction and ILC2 effector activity, whereas long-term benefits reflect deeper inhibition of remodeling pathways, reduced IgE-mediated amplification, and restoration of immune homeostasis [34].

This layered temporal pattern with a rapid symptomatic relief followed by slower structural normalization, helps explain the durability of dupilumab’s clinical response, as highlighted in the SINUS-24 and SINUS-52 trials [20,34]. Moreover, the dual impact on epithelial and innate compartments provides a mechanistic rationale for investigating baseline ILC2 phenotypes as potential biomarkers of therapeutic responsiveness. Altogether, IL-4Rα blockade emerges as a uniquely comprehensive strategy capable of reshaping both immune and structural components of type 2 nasal inflammation [158].

### 3.2. Dupilumab Effects on Nasal ILCs

Dupilumab exerts a coordinated immunoregulatory effect on the nasal mucosa by interrupting IL-4/IL-13–dependent pathways that sustain chronic ILC2 activation. In nasal tissue and peripheral blood, treatment is associated with a rapid decline in activated ILC2s, reflected by reduced expression of CRTH2, CD69 and GATA-3 and by diminished secretion of IL-5 and IL-13—key mediators of eosinophilic inflammation and mucus hyperproduction [19,21,137]. These early immunological shifts mirror the prompt disruption of the epithelial–ILC2 feedback loop that drives persistent type 2 inflammation. A comprehensive overview of dupilumab-mediated effects on nasal innate lymphoid cells, including mechanisms and clinical consequences, is provided in Table 1.

At the epithelial level, transcriptomic analyses demonstrate that IL-4Rα blockade promotes epithelial barrier restoration by upregulating genes involved in junctional integrity and ciliogenesis, while simultaneously reducing epithelial alarmins and chemokines that fuel innate activation [24,146]. This early stabilization of the epithelial barrier attenuates upstream inflammatory stimuli and contributes to the progressive functional quiescence of local ILC2s.

Beyond immediate effector suppression, dupilumab also influences the transcriptional and epigenetic programs governing ILC2 identity. Studies on ILC2 plasticity show that chronic exposure to inflammatory cytokines can drive the emergence of unstable intermediate phenotypes—hybrid ILC2/ILC1 states—associated with disease severity and reduced responsiveness to therapy. IL-4/IL-13 signaling plays a central role in maintaining chromatin accessibility at type 2 cytokine loci and in reinforcing effector polarization. Thus, IL-4Rα blockade reduces accessibility to these loci and stabilizes GATA-3-driven transcriptional programs. By limiting stress-induced plasticity, dupilumab helps preserve a more homeostatic and less pathogenic ILC2 phenotype [42,91,96,97].

A recent translational study by Golebski and colleagues [40] provided critical insight into how baseline innate lymphoid cell composition influences dupilumab responsiveness in CRSwNP. In a cohort of 38 CRSwNP patients treated with dupilumab, high-dimensional flow cytometry revealed that individuals with elevated baseline frequencies of inflammatory ILC2s (CD45RO^+^, CD62L^−^) were fast clinical responders, whereas slow responders showed increased proportions of ILC3s. Inflammatory ILC2s, derived from resting CD45RA^+^ ILC2s under mucosal cytokine stimulation, were enriched in patients who rapidly improved, suggesting that a strongly T2-skewed yet targetable immune landscape predicts greater treatment sensitivity. Functional assays confirmed that dupilumab directly suppressed IL-5 and IL-13 production by ILC2s in vitro without affecting their activation or differentiation state, demonstrating that IL-4Rα blockade primarily curtails cytokine effector output rather than cellular conversion. The authors proposed that baseline frequencies of inflammatory ILC2s may serve as predictive biomarkers of dupilumab efficacy and response speed, linking the therapy’s clinical benefits to its capacity to limit IL-13-mediated epithelial and eosinophilic inflammation. This study is particularly important because it connects clinical trajectories to precise innate immune phenotypes, underscoring the possibility of stratifying CRSwNP patients according to ILC-based biomarkers.

These findings reinforce the concept that dupilumab acts not only as a cytokine inhibitor but also as a modulator of innate immune dynamics, recalibrating the epithelial–ILC axis toward homeostasis. The early symptomatic improvements observed in fast responders likely reflect the rapid reduction of IL-13-driven inflammation and epithelial dysfunction. Moreover, by attenuating IL-33 and TSLP release from epithelial cells, dupilumab disrupts the amplification loop that perpetuates ILC2 activation, allowing restoration of mucosal integrity. This break in feedback signaling appears to be one of the pivotal steps in shifting the nasal environment from a perpetually activated state toward a more quiescent tissue profile [40].

Importantly, the profound reduction of epithelial alarmin release and type 2 inflammatory mediators also affects the behavior of nasal ILC progenitors (ILCp). Emerging evidence suggests that IL-4/IL-13 blockade decreases local differentiation signals that favor the continuous generation of inflammatory ILC2s from resident ILCp, thereby limiting the renewal of pathogenic effector subsets within the mucosa. This observation provides an explanation for the sustained benefit of dupilumab even in long-standing disease, where constant replenishment of inflammatory ILC2s contributes to chronicity and recurrence [40].

Further evidence of dupilumab’s immunomodulatory role at the systemic level was provided by Matsuyama and colleagues [19], who evaluated circulating immune profiles in CRSwNP patients before and after 24 weeks of therapy. Their study demonstrated a significant and sustained decrease in peripheral ILC2 frequencies following dupilumab treatment, paralleled by reductions in Th2 cells and serum IL-5 and IL-13 concentrations. The Th2/Th1 ratio markedly declined, while regulatory T cells showed a mild compensatory increase, suggesting a shift toward immune equilibrium. Notably, the decline in ILC2s correlated with both Th2 suppression and clinical improvement, indicating that IL-4Rα blockade exerts coordinated inhibition of innate and adaptive type 2 immunity. These systemic data reinforce the concept that dupilumab’s impact extends beyond local mucosal lesions, contributing to a global rebalancing of type 2 immunity [19].

Collectively, these mechanistic studies position dupilumab as a dual-level immunologic modulator, one that rapidly neutralizes type 2 cytokine signaling while progressively reprogramming both innate and adaptive immune compartments. By targeting the shared IL-4/IL-13 receptor, dupilumab interrupts the epithelial–ILC–Th2 feedback loop central to CRSwNP pathogenesis, leading to durable disease control and mucosal homeostasis. The identification of inflammatory ILC2s as both effectors and biomarkers of treatment response opens the way for personalized therapeutic strategies and real-time monitoring of biologic efficacy in type 2 inflammatory diseases of the upper airway. This perspective aligns with a broader shift in the field toward precision immunology, where innate immune profiling may soon guide both treatment selection and prediction of response dynamics.

Taken together, these findings highlight the central importance of ILC2s, along with their progenitors and transitional states, as both therapeutic targets and potential biomarkers of treatment responsiveness, opening the way for increasingly personalized approaches to biologic therapy in nasal type 2 inflammatory disease. As research continues to refine our understanding of ILC behavior, dupilumab may represent not only an effective therapeutic option but also a model for how targeted cytokine blockade can reset dysregulated innate immune networks in chronic airway disease.

## 4. Toward Precision Medicine: Integrating ILC Biology into Personalized Treatment Strategies

The rapidly expanding understanding of ILC biology and epithelial–immune crosstalk in the nasal mucosa is reshaping the way type 2 inflammatory diseases are defined, stratified, and treated [40,42,145]. Rather than viewing CRSwNP and severe allergic rhinitis as uniform clinical entities, current evidence reveals a constellation of distinct immunological endotypes, each driven by a characteristic pattern of epithelial activation, alarmin release, ILC subset composition, and downstream Th2 polarization [42,91,96,135,159]. This evolving framework is accelerating the transition from conventional symptom-oriented care toward precision immunomodulation, in which biologic therapy is tailored to the dominant inflammatory circuits operating in each patient [40,145].

A growing body of translational research indicates that the baseline architecture of the innate immune compartment profoundly shapes therapeutic responsiveness. Patients with a high burden of activated or inflammatory ILC2s, or with a strongly type-2-skewed ILC profile, frequently experience faster and more robust responses to IL-4Rα blockade. By contrast, individuals with higher proportions of ILC3s, mixed ILC2/ILC1 states, or signatures of ILC plasticity may require longer treatment durations or may benefit from alternative upstream strategies, particularly in cases where epithelium-derived alarmins remain the primary drivers of inflammation. These findings underscore the potential of ILC-centric immune profiling—including inflammatory vs. resting ILC2 states, ILC3-to-ILC1 ratios, and global ILC abundance—as a biologically grounded tool for meaningful patient stratification [42,91,96].

**Figure 4:**
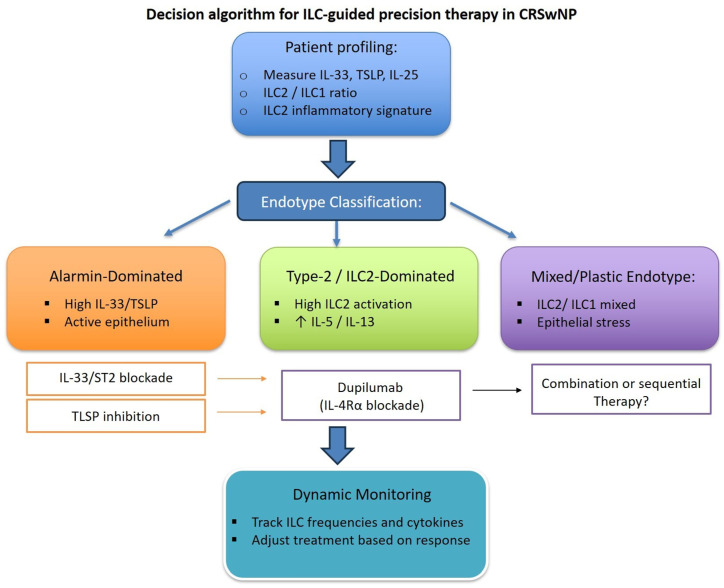
Proposed algorithm for precision-medicine strategies in CRSwNP integrating ILC biology. Patient stratification combines epithelial-derived alarmin expression (IL-33, TSLP), ILC subset profiling (ILC2/ILC1 ratios, inflammatory ILC2 signatures), and clinical phenotypes to guide biologic therapy. High alarmin/endotype patients may benefit from upstream IL-33 or TSLP blockade, whereas type-2/ILC2-dominant patients are likely to respond rapidly to downstream IL-4Rα inhibition (dupilumab). Mixed or plastic endotypes may require sequential or combination approaches. Longitudinal monitoring of immune markers allows real-time adjustment and optimization of therapy.

In parallel, epithelial biology is emerging as an equally powerful determinant of treatment choice. Elevated IL-33 or TSLP expression, persistent epithelial stress responses, and structural barrier defects define patients with alarmin-dominated disease, a phenotype that may be optimally addressed through targeted IL-33/ST2 or TSLP inhibition. Conversely, patients with modest alarmin elevation but strong type-2 cytokine output may benefit most from dupilumab as a first-line biologic, reflecting a downstream blockade approach within the epithelial–ILC–Th2 axis. In this context, an algorithmic strategy integrating epithelial alarmin levels, ILC subset composition (including ILC2/ILC1 balance and inflammatory ILC2 signatures), and downstream inflammatory markers may help translate immunological complexity into clinically actionable treatment selection, providing a framework for precision medicine in type 2 upper airway disease (as shown in Figure 4).

This concept of endotype-directed therapeutic sequencing—intervening upstream or downstream according to the predominant inflammatory pathway—represents a rational and increasingly evidence-supported strategy for optimizing clinical outcomes [137,145,160].

Precision medicine also extends beyond baseline stratification to include longitudinal immune monitoring. Peripheral and tissue-resident ILC2 frequencies, nasal cytokine patterns, epithelial barrier biomarkers, transcriptional states, and even epigenetic signatures of ILC activation are emerging as dynamic readouts of treatment efficacy. Integrating these markers with high-dimensional technologies such as mass cytometry, single-cell transcriptomics, proteomics, and functional epithelial assays may ultimately enable real-time adjustment of biologic therapy, ensuring durable disease control while avoiding unnecessary exposure to immunomodulation.

Future personalization strategies may further incorporate metabolic, neuroimmune, and microenvironmental determinants of ILC2 activation. Because ILC2s are highly sensitive to oxygen availability, nutrient gradients, neuronal mediators, and tissue-derived lipids, these cues may help refine dosing intervals, identify windows of heightened responsiveness, or guide the use of combination approaches that synergize with IL-4Rα blockade. Within this emerging paradigm, therapy may extend beyond monotherapy to coordinated regimens that simultaneously or sequentially target epithelial alarmins, ILC effector pathways, and the microenvironmental context that sustains type 2 inflammation [42,91,96].

**Figure 5:**
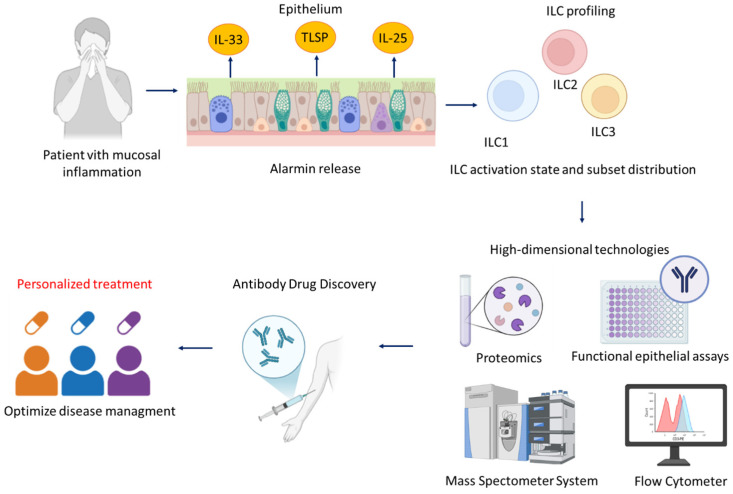
Schematic overview of ILC-driven endotypes and precision-medicine strategies in CRSwNP. This schematic summarizes how epithelial activation, alarmin release (IL-33, IL-25, TSLP), and the distribution of nasal ILC subsets—including inflammatory ILC2s, hybrid ILC2/ILC1 populations, ILC3-skewed states, and local ILC progenitors—converge to define distinct inflammatory endotypes in CRSwNP. These endotypes help predict responsiveness to targeted biologics: strong type-2/ILC2 dominance is associated with rapid improvement under IL-4Rα blockade (dupilumab), whereas endotypes characterized by high epithelial alarmin activity may benefit from upstream IL-33/ST2 or TSLP inhibition. The schematic also highlights emerging biomarkers—such as ILC subset frequencies, inflammatory ILC2 phenotypes, epithelial-derived alarmin signatures, and neuroimmune or metabolic cues—that support stratified therapeutic decision-making within precision-medicine frameworks. Created in BioRender. Chiarella, E. (2026) https://BioRender.com/r8zbfng (accessed on 11 February 2026).

Taken together, these insights mark a decisive shift toward a biologically anchored, patient-specific model of care. By integrating epithelial profiling, ILC subset analysis, and dynamic biomarker monitoring, the field moves closer to truly personalized therapy for CRSwNP and severe allergic rhinitis. Within this broader perspective, Figure 5 illustrates how current and emerging biologic therapies may be positioned along the epithelial–ILC–Th2 axis, emphasizing the continuum and plasticity of inflammatory pathways rather than a fixed treatment hierarchy. In this landscape, dupilumab represents not only a highly effective treatment but also a proof-of-concept model demonstrating how mechanistic understanding of the epithelial–ILC–Th2 axis can translate into precision interventions. Importantly, this conceptual framework is not limited to IL-4Rα-targeted therapy. Emerging biologic agents directed against epithelial-derived alarmins, particularly thymic stromal lymphopoietin (TSLP), may further expand precision medicine strategies by acting upstream at the level of epithelial–immune crosstalk. In patients with alarmin-dominated disease signatures, such approaches may complement or, in selected endotypes, represent alternatives to downstream cytokine blockade [161,162,163]. Ultimately, the expanding knowledge of ILC biology positions innate lymphoid cells at the center of next-generation, endotype-driven therapeutic strategies for type 2 inflammatory diseases of the upper airway.

## 5. Conclusions

Innate lymphoid cells, particularly ILC2s, emerge as central conductors of type 2 inflammation in the nasal mucosa, integrating signals from epithelial alarmins, neuronal pathways, metabolic cues, and stromal interactions into coordinated effector programs. Their strategic localization at epithelial barrier surfaces, rapid responsiveness to danger signals, and remarkable functional plasticity place them at the crossroads of tissue immunity, where they drive eosinophilic inflammation, mucus hypersecretion, and the progressive remodeling characteristic of chronic disease. Recent insights further reveal that both mature ILC2s and their resident progenitors contribute not only to disease initiation but also to its persistence and chronicity, sustaining inflammatory cycles that define CRSwNP and severe allergic rhinitis.

Dupilumab, through targeted IL-4Rα blockade, exemplifies how a biologic therapy grounded in mechanistic understanding can modulate interconnected innate and adaptive pathways. Clinical and translational studies consistently show that dupilumab reduces activated ILC2 populations, suppresses IL-5 and IL-13 secretion, restores epithelial barrier integrity, and disrupts the epithelial–ILC–Th2 amplification loop that underlies chronic type 2 inflammation. Meanwhile, emerging upstream therapies targeting IL-33 or TSLP provide complementary opportunities to intervene even earlier in the cascade of ILC activation, broadening the therapeutic armamentarium.

Looking ahead, the integration of high-dimensional immune profiling, epithelial barrier assessment, metabolic characterization, and ILC-focused biomarkers holds promise for a new era of deeply personalized immunomodulation. By uniting fundamental mechanistic insight with clinical precision, advances in nasal ILC biology offer a transformative framework for long-term management of type 2 inflammatory diseases of the upper airway.

## Figures and Tables

**Table 1:** Effects of Dupilumab on Nasal ILCs.

Biological Feature	Effect of Dupilumab	Mechanism	Clinical/Functional Consequence	Ref.
Activated ILC2s in nasal tissue and peripheral blood	Rapid reduction of activated ILC2s	Decreased CRTH2, CD69, GATA-3 expression; reduced IL-5 and IL-13 secretion	Reduced eosinophilic inflammation and mucus overproduction	[18,20,135]
Epithelial–ILC2 feedback loop	Rapid disruption	Blocks IL-4/IL-13-dependent pathways sustaining ILC2 activation	Local functional quiescence of ILC2s	[18,20,135]
Epithelial barrier integrity	Barrier restoration	Upregulates genes for junctional integrity and ciliogenesis; decreases alarmins and chemokines	Early barrier stabilization; reduces upstream inflammatory stimuli	[24,34]
ILC2 plasticity	Limits intermediate ILC2/ILC1 phenotypes	IL-4/IL-13 blockade reduces chromatin accessibility at type 2 cytokine loci; stabilizes GATA-3	Maintains a less pathogenic, homeostatic ILC2 phenotype	[40,89,93,94]
Predictors of response to dupilumab	High baseline inflammatory ILC2 → rapid clinical response	Inflammatory ILC2s (CD45RO^+^, CD62L^−^) derived from CD45RA^+^ precursors under mucosal cytokine stimulation	Potential predictive biomarkers for treatment speed and responsiveness	[40]
ILC2 cytokine production	Reduced IL-5 and IL-13 without affecting activation/differentiation	IL-4Rα blockade primarily limits cytokine effector output	Controls epithelial and eosinophilic inflammation	[40]
Epithelial alarmins (IL-33, TSLP)	Decreased release	IL-4/IL-13 blockade	Disruption of amplification loop; restoration of mucosal integrity	[35]
ILC progenitors (ILCp)	Reduced differentiation into inflammatory ILC2s	Local differentiation signals for inflammatory ILC2s diminished	Sustained long-term benefit; reduces chronicity and recurrence	[35]
Systemic effects on ILC2 and Th2	Decreased peripheral ILC2; lower Th2/Th1 ratio; mild increase in Tregs	Coordinated IL-4Rα inhibition	Global rebalancing of type 2 immunity; correlates with clinical improvement	[18]

Overview of dupilumab’s effects on nasal innate lymphoid cells (ILCs). The table outlines the main immune and tissue features affected by treatment, the specific changes observed (e.g., reduced ILC2 activation or cytokine release), and the underlying mechanisms linked to IL-4/IL-13 blockade. It also summarizes the clinical consequences of these effects, including decreased inflammation and improved epithelial barrier function.

## Data Availability

No new data were created or analyzed in this study.

## References

[1] Alfi O., Yakirevitch A., Wald O., Wandel O., Izhar U., Oiknine-Djian E., Nevo Y., Elgavish S., Dagan E., Madgar O. (2021). Human Nasal and Lung Tissues Infected Ex Vivo with SARS-CoV-2 Provide Insights into Differential Tissue-Specific and Virus-Specific Innate Immune Responses in the Upper and Lower Respiratory Tract. J. Virol..

[2] Zhang R., Zhang L., Li P., Pang K., Liu H., Tian L. (2023). Epithelial Barrier in the Nasal Mucosa, Related Risk Factors and Diseases. Int. Arch. Allergy Immunol..

[3] Jong R.M., Van Dis E., Berry S.B., Nguyenla X., Baltodano A., Pastenkos G., Xu C., Fox D., Yosef N., McWhirter S.M. (2022). Mucosal Vaccination with Cyclic Dinucleotide Adjuvants Induces Effective T Cell Homing and IL-17–Dependent Protection against Mycobacterium Tuberculosis Infection. J. Immunol..

[4] Li M., Gan C., Zhang R., Wang J., Wang Y., Zhu W., Liu L., Shang J., Zhao Q. (2023). TRAF5 Regulates Intestinal Mucosal Th1/Th17 Cell Immune Responses via Runx1 in Colitis Mice. Immunology.

[5] Brockmann L., Tran A., Huang Y., Edwards M., Ronda C., Wang H.H., Ivanov I.I. (2023). Intestinal Microbiota-Specific Th17 Cells Possess Regulatory Properties and Suppress Effector T Cells via c-MAF and IL-10. Immunity.

[6] Piazzetta G.L., Lobello N., Pelaia C., Mariaimmacolata P., Lombardo N., Chiarella E. (2025). Modulating Nasal Barrier Function and Tissue Remodeling in Inflammatory Diseases: The Role of Ginseng and Its Bioactive Compounds. Tissue Barriers.

[7] Ryu S., Lim M.Y., Kim J., Kim H.Y. (2023). Versatile Roles of Innate Lymphoid Cells at the Mucosal Barrier: From Homeostasis to Pathological Inflammation. Exp. Mol. Med..

[8] Fan H., Wang A., Wang Y., Sun Y., Han J., Chen W., Wang S., Wu Y., Lu Y. (2019). Innate Lymphoid Cells: Regulators of Gut Barrier Function and Immune Homeostasis. J. Immunol. Res..

[9] Fol M., Karpik W., Zablotni A., Kulesza J., Kulesza E., Godkowicz M., Druszczynska M. (2024). Innate Lymphoid Cells and Their Role in the Immune Response to Infections. Cells.

[10] Vivier E., Artis D., Colonna M., Diefenbach A., Di Santo J.P., Eberl G., Koyasu S., Locksley R.M., McKenzie A.N.J., Mebius R.E. (2018). Innate Lymphoid Cells: 10 Years on. Cell.

[11] Monticelli L.A., Sonnenberg G.F., Abt M.C., Alenghat T., Ziegler C.G.K., Doering T.A., Angelosanto J.M., Laidlaw B.J., Yang C.Y., Sathaliyawala T. (2011). Innate Lymphoid Cells Promote Lung-Tissue Homeostasis after Infection with Influenza Virus. Nat. Immunol..

[12] Van Acker N., Frenois F.X., Gravelle P., Tosolini M., Syrykh C., Laurent C., Brousset P. (2025). Spatial Mapping of Innate Lymphoid Cells in Human Lymphoid Tissues and Lymphoma at Single-Cell Resolution. Nat. Commun..

[13] Wang S., Liu X., Lin X., Lv X., Zhang H. (2024). Group 2 Innate Lymphoid Cells in Allergic Rhinitis. J. Inflamm. Res..

[14] Ho J., Bailey M., Zaunders J., Mrad N., Sacks R., Sewell W., Harvey R.J. (2015). Group 2 Innate Lymphoid Cells (ILC2s) Are Increased in Chronic Rhinosinusitis with Nasal Polyps or Eosinophilia. Clin. Exp. Allergy.

[15] Moro K., Yamada T., Tanabe M., Takeuchi T., Ikawa T., Kawamoto H., Furusawa J.I., Ohtani M., Fujii H., Koyasu S. (2010). Innate Production of T(H)2 Cytokines by Adipose Tissue-Associated c-Kit(+)Sca-1(+) Lymphoid Cells. Nature.

[16] Maspero J., Adir Y., Al-Ahmad M., Celis-Preciado C.A., Colodenco F.D., Giavina-Bianchi P., Lababidi H., Ledanois O., Mahoub B., Perng D.W. (2022). Type 2 Inflammation in Asthma and Other Airway Diseases. ERJ Open Res..

[17] Yin Z., Zhou Y., Turnquist H.R., Liu Q. (2022). Neuro–Epithelial–ILC2 Crosstalk in Barrier Tissues. Trends Immunol..

[18] Naito M., Kumanogoh A. (2023). Group 2 Innate Lymphoid Cells and Their Surrounding Environment. Inflamm. Regen..

[19] Matsuyama T., Takahashi H., Tada H., Chikamatsu K. (2023). Circulating T Cell Subsets and ILC2s Are Altered in Patients with Chronic Rhinosinusitis with Nasal Polyps After Dupilumab Treatment. Am. J. Rhinol. Allergy.

[20] Bachert C., Laidlaw T.M., Cho S.H., Mullol J., Swanson B.N., Naimi S., Classe M., Harel S., Jagerschmidt A., Laws E. (2023). Effect of Dupilumab on Type 2 Biomarkers in Chronic Rhinosinusitis with Nasal Polyps: SINUS-52 Study Results. Ann. Otol. Rhinol. Laryngol..

[21] Jonstam K., Swanson B.N., Mannent L.P., Cardell L.O., Tian N., Wang Y., Zhang D., Fan C., Holtappels G., Hamilton J.D. (2019). Dupilumab Reduces Local Type 2 Pro-Inflammatory Biomarkers in Chronic Rhinosinusitis with Nasal Polyposis. Allergy.

[22] Ryser F.S., Demeter T., Pijuan J.B., Shambat S.M., Brühlmann C., Mauthe T., Hilty M., Soyka M.B., Steiner U.C., Brugger S.D. (2025). Dupilumab Treatment Is Associated with Clinical Improvement and a Shift Toward a Health-Associated Nasal Passage Microbiota in Diffuse Type 2 Chronic Rhinosinusitis. Allergy.

[23] Piazzetta G.L., Lobello N., Chiarella E., Rizzuti A., Pelaia C., Pelaia G., Lombardo N. (2023). Targeting IL-4 and IL-13 Receptors on Eosinophils in CRSwNP Patients: The Clinical Efficacy of Dupilumab. J. Pers. Med..

[24] Fieux M., Carsuzaa F., Bellanger Y., Bartier S., Fournier V., Lecron J.C., Bainaud M., Louis B., Tringali S., Dufour X. (2024). Dupilumab Prevents Nasal Epithelial Function Alteration by IL-4 in Vitro: Evidence for Its Efficacy. Int. Forum Allergy Rhinol..

[25] Albrecht T., Sailer M.M., Capitani F., van Schaik C., Löwenheim H., Becker S. (2023). Real-World Evidence for the Effectiveness and Safety of Dupilumab in Patients with CRSwNP after 1 Year of Therapy. World Allergy Organ. J..

[26] Rodriguez-Iglesias M., Calvo-Henríquez C., Martin-Jimenez D., García-Lliberós A., Maza-Solano J., Moreno-Luna R., Izquierdo-Domínguez A., Martínez-Capoccioni G., Alobid I. (2025). Effect of Dupilumab in CRSwNP Sinonasal Outcomes from Real Life Studies: A Systematic Review with Meta-Analysis. Curr. Allergy Asthma Rep..

[27] Nettis E., Brussino L., Patella V., Bonzano L., Detoraki A., Di Leo E., Sirufo M.M., Caruso C., Lodi Rizzini F., Conte M. (2022). Effectiveness and Safety of Dupilumab in Patients with Chronic Rhinosinusitis with Nasal Polyps and Associated Comorbidities: A Multicentric Prospective Study in Real Life. Clin. Mol. Allergy.

[28] Boesjes C.M., Van Der Gang L.F., Bakker D.S., Ten Cate T.A., Spekhorst L.S., De Graaf M., Van Dijk M.R., De Bruin-Weller M.S. (2023). Dupilumab-Associated Lymphoid Reactions in Patients with Atopic Dermatitis. JAMA Dermatol..

[29] Hasan I., Parsons L., Duran S., Zinn Z. (2024). Dupilumab Therapy for Atopic Dermatitis Is Associated with Increased Risk of Cutaneous T Cell Lymphoma: A Retrospective Cohort Study. J. Am. Acad. Dermatol..

[30] Li M., Zhao W., Lai P., Xiao Y., Wang Y. (2025). Dupilumab-Associated Lymphoproliferative Disorders: A Comprehensive Review on Clinicohistopathologic Features and Underlying Mechanisms. Curr. Opin. Immunol..

[31] Lavin L., Geller S. (2025). Cutaneous T Cell Lymphoma Following Dupilumab Therapy in Patients with Atopic Dermatitis: Clinical Review and Recommendations. Am. J. Clin. Dermatol..

[32] Chiarella E., Lombardo N., Lobello N., Aloisio A., Aragona T., Pelaia C., Scicchitano S., Bond H.M., Mesuraca M. (2020). Nasal Polyposis: Insights in Epithelial-Mesenchymal Transition and Differentiation of Polyp Mesenchymal Stem Cells. Int. J. Mol. Sci..

[33] Kotas M.E., Patel N.N., Cope E.K., Gurrola J.G., Goldberg A.N., Pletcher S.D., Seibold M.A., Moore C.M., Gordon E.D. (2023). IL-13–Associated Epithelial Remodeling Correlates with Clinical Severity in Nasal Polyposis. J. Allergy Clin. Immunol..

[34] Bachert C., Hicks A., Gane S., Peters A.T., Gevaert P., Nash S., Horowitz J.E., Sacks H., Jacob-Nara J.A. (2024). The Interleukin-4/Interleukin-13 Pathway in Type 2 Inflammation in Chronic Rhinosinusitis with Nasal Polyps. Front. Immunol..

[35] Stevens W.W., Kato A. (2020). Group 2 Innate Lymphoid Cells in Nasal Polyposis. Ann. Allergy Asthma Immunol..

[36] Artis D., Spits H. (2015). The Biology of Innate Lymphoid Cells. Nature.

[37] Spits H., Artis D., Colonna M., Diefenbach A., Di Santo J.P., Eberl G., Koyasu S., Locksley R.M., McKenzie A.N.J., Mebius R.E. (2013). Innate Lymphoid Cells—A Proposal for Uniform Nomenclature. Nat. Rev. Immunol..

[38] Bennstein S.B., Uhrberg M. (2022). Biology and Therapeutic Potential of Human Innate Lymphoid Cells. FEBS J..

[39] Thomas C.M., Peebles R.S. (2022). Development and Function of Regulatory Innate Lymphoid Cells. Front. Immunol..

[40] Golebski K., van der Lans R.J.L., van Egmond D., de Groot E., Spits H., van der Zee A.H.M., van Drunen C.M., Fokkens W.J., Reitsma S. (2023). Inflammatory Innate Lymphoid Cells Predict Response Speed to Dupilumab in Chronic Rhinosinusitis with Nasal Polyps. Allergy Eur. J. Allergy Clin. Immunol..

[41] Kim D.H., Lim J.Y., Jang J.Y., Gwak J., Joo H.A., Ryu S., Kim J.H. (2023). Distinct Subsets of Innate Lymphoid Cells in Nasal Polyp. Allergol. Int..

[42] Korchagina A.A., Shein S.A., Koroleva E., Tumanov A.V. (2023). Transcriptional Control of ILC Identity. Front. Immunol..

[43] Lopes N., Galluso J., Escalière B., Carpentier S., Kerdiles Y.M., Vivier E. (2022). Tissue-Specific Transcriptional Profiles and Heterogeneity of Natural Killer Cells and Group 1 Innate Lymphoid Cells. Cell Rep. Med..

[44] Thio C.L.P., Shao J.S., Luo C.H., Chang Y.J. (2025). Decoding Innate Lymphoid Cells and Innate-like Lymphocytes in Asthma: Pathways to Mechanisms and Therapies. J. Biomed. Sci..

[45] Jia H., Wan H., Zhang D. (2023). Innate Lymphoid Cells: A New Key Player in Atopic Dermatitis. Front. Immunol..

[46] Berkinbayeva M., Gu W., Chen Z., Gao P. (2024). Group 3 Innate Lymphoid Cells: A Potential Therapeutic Target for Steroid Resistant Asthma. Clin. Rev. Allergy Immunol..

[47] Seillet C., Brossay L., Vivier E. (2020). Natural Killers or ILC1s? That Is the Question. Curr. Opin. Immunol..

[48] Ghaedi M., Takei F. (2021). Innate Lymphoid Cell Development. J. Allergy Clin. Immunol..

[49] Liu C., Gong Y., Zhang H., Yang H., Zeng Y., Bian Z., Xin Q., Bai Z., Zhang M., He J. (2021). Delineating Spatiotemporal and Hierarchical Development of Human Fetal Innate Lymphoid Cells. Cell Res..

[50] Ding Y., Harly C., Das A., Bhandoola A. (2023). Early Development of Innate Lymphoid Cells. Methods Mol. Biol..

[51] Scoville S.D., Freud A.G., Caligiuri M.A. (2018). Cellular Pathways in the Development of Human and Murine Innate Lymphoid Cells. Curr. Opin. Immunol..

[52] Yang Q., Li F., Harly C., Xing S., Ye L., Xia X., Wang H., Wang X., Yu S., Zhou X. (2015). TCF-1 Upregulation Identifies Early Innate Lymphoid Progenitors in the Bone Marrow. Nat. Immunol..

[53] Ren G., Lai B., Harly C., Baek S., Ding Y., Zheng M., Cao Y., Cui K., Yang Y., Zhu J. (2022). Transcription Factors TCF-1 and GATA3 Are Key Factors for the Epigenetic Priming of Early Innate Lymphoid Progenitors toward Distinct Cell Fates. Immunity.

[54] Zhong C., Zheng M., Cui K., Martins A.J., Hu G., Li D., Tessarollo L., Kozlov S., Keller J.R., Tsang J.S. (2020). Differential Expression of the Transcription Factor GATA3 Specifies Lineage and Functions of Innate Lymphoid Cells. Immunity.

[55] Yu X., Wang Y., Deng M., Li Y., Ruhn K.A., Zhang C.C., Hooper L.V. (2014). The Basic Leucine Zipper Transcription Factor NFIL3 Directs the Development of a Common Innate Lymphoid Cell Precursor. eLife.

[56] Stehle C., Rückert T., Fiancette R., Gajdasik D.W., Willis C., Ulbricht C., Durek P., Mashreghi M.F., Finke D., Hauser A.E. (2021). T-Bet and RORα Control Lymph Node Formation by Regulating Embryonic Innate Lymphoid Cell Differentiation. Nat. Immunol..

[57] Kasal D.N., Bendelac A. (2020). Multi-Transcription Factor Reporter Mice Delineate Early Precursors to the ILC and LTi Lineages. J. Exp. Med..

[58] Irie M., Kabata H., Fukunaga K. (2024). Protocol for Lentiviral Vector-Based Gene Transfection in Human ILC2s. STAR Protoc..

[59] Chiarella E., Carrá G., Scicchitano S., Codispoti B., Mega T., Lupia M., Pelaggi D., Marafioti M.G., Aloisio A., Giordano M. (2014). UMG Lenti: Novel Lentiviral Vectors for Efficient Transgene- and Reporter Gene Expression in Human Early Hematopoietic Progenitors. PLoS ONE.

[60] Huang J., Long Z., Jia R., Wang M., Zhu D., Liu M., Chen S., Zhao X., Yang Q., Wu Y. (2021). The Broad Immunomodulatory Effects of IL-7 and Its Application in Vaccines. Front. Immunol..

[61] Calvi M., Di Vito C., Frigo A., Trabanelli S., Jandus C., Mavilio D. (2022). Development of Human ILCs and Impact of Unconventional Cytotoxic Subsets in the Pathophysiology of Inflammatory Diseases and Cancer. Front. Immunol..

[62] Chen D., Tang T.X., Deng H., Yang X.P., Tang Z.H. (2021). Interleukin-7 Biology and Its Effects on Immune Cells: Mediator of Generation, Differentiation, Survival, and Homeostasis. Front. Immunol..

[63] Klose C.S.N., Blatz K., d’Hargues Y., Hernandez P.P., Kofoed-Nielsen M., Ripka J.F., Ebert K., Arnold S.J., Diefenbach A., Palmer E. (2014). The Transcription Factor T-Bet Is Induced by IL-15 and Thymic Agonist Selection and Controls CD8αα+ Intraepithelial Lymphocyte Development. Immunity.

[64] Berjis A., Muthumani D., Aguilar O.A., Pomp O., Johnson O., Finck A.V., Engel N.W., Chen L., Plachta N., Scholler J. (2024). Pretreatment with IL-15 and IL-18 Rescues Natural Killer Cells from Granzyme B-Mediated Apoptosis after Cryopreservation. Nat. Commun..

[65] Klose C.S.N., Artis D. (2020). Innate Lymphoid Cells Control Signaling Circuits to Regulate Tissue-Specific Immunity. Cell Res..

[66] Kasal D.N., Liang Z., Hollinger M.K., O’Leary C.Y., Lisicka W., Sperling A.I., Bendelac A. (2021). A Gata3 Enhancer Necessary for ILC2 Development and Function. Proc. Natl. Acad. Sci. USA.

[67] Ferreira A.C.F., Szeto A.C.H., Heycock M.W.D., Clark P.A., Walker J.A., Crisp A., Barlow J.L., Kitching S., Lim A., Gogoi M. (2021). RORα Is a Critical Checkpoint for T Cell and ILC2 Commitment in the Embryonic Thymus. Nat. Immunol..

[68] Klein Wolterink R.G.J., Serafini N., Van Nimwegen M., Vosshenrich C.A.J., De Bruijn M.J.W., Pereira D.F., Fernandes H.V., Hendriks R.W., Di Santo J.P. (2013). Essential, Dose-Dependent Role for the Transcription Factor Gata3 in the Development of IL-5+ and IL-13+ Type 2 Innate Lymphoid Cells. Proc. Natl. Acad. Sci. USA.

[69] Withers D.R., Hepworth M.R., Wang X., Mackley E.C., Halford E.E., Dutton E.E., Marriott C.L., Brucklacher-Waldert V., Veldhoen M., Kelsen J. (2016). Transient Inhibition of ROR-Γt Therapeutically Limits Intestinal Inflammation by Reducing TH17 Cells and Preserving ILC3. Nat. Med..

[70] Lv X., Zhu S., Wu J., Chen J. (2022). Transcriptional Control of Mature ILC3 Function and Plasticity: Not Just RORγt. Cell. Mol. Immunol..

[71] Mowel W.K., McCright S.J., Kotzin J.J., Collet M.A., Uyar A., Chen X., DeLaney A., Spencer S.P., Virtue A.T., Yang E.J. (2017). Group 1 Innate Lymphoid Cell Lineage Identity Is Determined by a Cis-Regulatory Element Marked by a Long Non-Coding RNA. Immunity.

[72] Klose C.S.N., Flach M., Möhle L., Rogell L., Hoyler T., Ebert K., Fabiunke C., Pfeifer D., Sexl V., Fonseca-Pereira D. (2014). Differentiation of Type 1 ILCs from a Common Progenitor to All Helper-like Innate Lymphoid Cell Lineages. Cell.

[73] Zhou W., Sonnenberg G.F. (2020). Activation and Suppression of Group 3 Innate Lymphoid Cells in the Gut. Trends Immunol..

[74] Murphy J.M., Ngai L., Mortha A., Crome S.Q. (2022). Tissue-Dependent Adaptations and Functions of Innate Lymphoid Cells. Front. Immunol..

[75] Walker J.A., Clark P.A., Crisp A., Barlow J.L., Szeto A., Ferreira A.C.F., Rana B.M.J., Jolin H.E., Rodriguez-Rodriguez N., Sivasubramaniam M. (2019). Polychromic Reporter Mice Reveal Unappreciated Innate Lymphoid Cell Progenitor Heterogeneity and Elusive ILC3 Progenitors in Bone Marrow. Immunity.

[76] Lim A.I., Di Santo J.P. (2019). ILC-Poiesis: Ensuring Tissue ILC Differentiation at the Right Place and Time. Eur. J. Immunol..

[77] Hernández-Torres D.C., Stehle C. (2022). Embryonic ILC-Poiesis across Tissues. Front. Immunol..

[78] Liu Q., Lee J.H., Kang H.M., Kim C.H. (2022). Identification of the Niche and Mobilization Mechanism for Tissue-Protective Multipotential Bone Marrow ILC Progenitors. Sci. Adv..

[79] Zeis P., Lian M., Fan X., Herman J.S., Hernandez D.C., Gentek R., Elias S., Symowski C., Knöpper K., Peltokangas N. (2020). In Situ Maturation and Tissue Adaptation of Type 2 Innate Lymphoid Cell Progenitors. Immunity.

[80] Dhariwal J., Cameron A., Trujillo-Torralbo M.B., Del Rosario A., Bakhsoliani E., Paulsen M., Jackson D.J., Edwards M.R., Rana B.M.J., Cousins D.J. (2017). Mucosal Type 2 Innate Lymphoid Cells Are a Key Component of the Allergic Response to Aeroallergens. Am. J. Respir. Crit. Care Med..

[81] Keir M.E., Yi T., Lu T.T., Ghilardi N. (2020). The Role of IL-22 in Intestinal Health and Disease. J. Exp. Med..

[82] Shannon J.P., Vrba S.M., Reynoso G.V., Wynne-Jones E., Kamenyeva O., Malo C.S., Cherry C.R., McManus D.T., Hickman H.D. (2021). Group 1 Innate Lymphoid Cell-Derived Interferon-γ Maintains Anti-Viral Vigilance in the Mucosal Epithelium. Immunity.

[83] Kato A. (2019). Group 2 Innate Lymphoid Cells in Airway Diseases. Chest.

[84] Heinrich B., Korangy F. (2022). Plasticity of Innate Lymphoid Cells in Cancer. Front. Immunol..

[85] Schulz-Kuhnt A., Wirtz S., Neurath M.F., Atreya I. (2020). Regulation of Human Innate Lymphoid Cells in the Context of Mucosal Inflammation. Front. Immunol..

[86] Shao F., Yu D., Xia P., Wang S. (2021). Dynamic Regulation of Innate Lymphoid Cells in the Mucosal Immune System. Cell. Mol. Immunol..

[87] Lim A.I., Menegatti S., Bustamante J., Le Bourhis L., Allez M., Rogge L., Casanova J.L., Yssel H., Di Santo J.P. (2016). IL-12 Drives Functional Plasticity of Human Group 2 Innate Lymphoid Cells. J. Exp. Med..

[88] Emami Fard N., Xiao M., Sehmi R. (2023). Regulatory ILC2—Role of IL-10 Producing ILC2 in Asthma. Cells.

[89] Nagasawa M., Spits H., Romero Ros X. (2018). Innate Lymphoid Cells (ILCs): Cytokine Hubs Regulating Immunity and Tissue Homeostasis. Cold Spring Harb. Perspect. Biol..

[90] Cobb L.M., Verneris M.R. (2021). Therapeutic Manipulation of Innate Lymphoid Cells. JCI Insight.

[91] Kabil A., Shin S.B., Hughes M.R., McNagny K.M. (2022). “Just One Word, Plastic!”: Controversies and Caveats in Innate Lymphoid Cell Plasticity. Front. Immunol..

[92] Cheng H., Jin C., Wu J., Zhu S., Liu Y.J., Chen J. (2017). Guards at the Gate: Physiological and Pathological Roles of Tissue-Resident Innate Lymphoid Cells in the Lung. Protein Cell.

[93] Huang Y.-A., Wang X., Kim J.-C., Yao X., Sethi A., Strohm A., Doherty T.A. (2024). PIP-Seq Identifies Novel Heterogeneous Lung Innate Lymphocyte Population Activation after Combustion Product Exposure. Sci. Rep..

[94] De Salvo C., Buela K.A., Pizarro T.T. (2020). Cytokine-Mediated Regulation of Innate Lymphoid Cell Plasticity in Gut Mucosal Immunity. Front. Immunol..

[95] Camiolo M.J., Zhou X., Oriss T.B., Yan Q., Gorry M., Horne W., Trudeau J.B., Scholl K., Chen W., Kolls J.K. (2021). High-Dimensional Profiling Clusters Asthma Severity by Lymphoid and Non-Lymphoid Status. Cell Rep..

[96] Verma M., McKay J., Verma D. (2023). Role of Epigenetics in Innate Lymphoid Cells. Epigenomics.

[97] Sun H., Qiu J., Qiu J. (2024). Epigenetic Regulation of Innate Lymphoid Cells. Eur. J. Immunol..

[98] Galle-Treger L., Hurrell B.P., Lewis G., Howard E., Jahani P.S., Banie H., Razani B., Soroosh P., Akbari O. (2020). Autophagy Is Critical for Group 2 Innate Lymphoid Cell Metabolic Homeostasis and Effector Function. J. Allergy Clin. Immunol..

[99] Michaeloudes C., Bhavsar P.K., Mumby S., Xu B., Hui C.K.M., Chung K.F., Adcock I.M. (2020). Role of Metabolic Reprogramming in Pulmonary Innate Immunity and Its Impact on Lung Diseases. J. Innate Immun..

[100] Pelletier A., Stockmann C. (2022). The Metabolic Basis of ILC Plasticity. Front. Immunol..

[101] Ham J., Yang W., Kim H.Y. (2025). Tissue-Specific Metabolic Reprogramming in Innate Lymphoid Cells and Its Impact on Disease. Immune Netw..

[102] Yu H., Jacquelot N., Belz G.T. (2022). Metabolic Features of Innate Lymphoid Cells. J. Exp. Med..

[103] Zhang X., Liu J., Li X., Zheng G., Wang T., Sun H., Huang Z., He J., Qiu J., Zhao Z. (2025). Blocking the HIF-1α/Glycolysis Axis Inhibits Allergic Airway Inflammation by Reducing ILC2 Metabolism and Function. Allergy.

[104] Zhou L., Lin Q., Sonnenberg G.F. (2022). Metabolic Control of Innate Lymphoid Cells in Health and Disease. Nat. Metab..

[105] Olsthoorn S.E.M., van Krimpen A., Hendriks R.W., Stadhouders R. (2025). Chronic Inflammation in Asthma: Looking Beyond the Th2 Cell. Immunol Rev..

[106] Izumi M., Nakanishi Y., Kang S., Kumanogoh A. (2024). Peripheral and Central Regulation of Neuro–Immune Crosstalk. Inflamm. Regen..

[107] Liu W., Wang S., Wang J., Zheng R., Wang D., Yu R., Liu B. (2023). Neuromedin U Induces Pulmonary ILC2 Activation via the NMUR1 Pathway during Acute Respiratory Syncytial Virus Infection. Am. J. Respir. Cell Mol. Biol..

[108] Pascal M., Kazakov A., Chevalier G., Dubrule L., Deyrat J., Dupin A., Saha S., Jagot F., Sailor K., Dulauroy S. (2022). The Neuropeptide VIP Potentiates Intestinal Innate Type 2 and Type 3 Immunity in Response to Feeding. Mucosal Immunol..

[109] Pinho-Ribeiro F.A., Verri W.A., Chiu I.M. (2017). Feature Review Nociceptor Sensory Neuron-Immune Interactions in Pain and Inflammation. Trends Immunol..

[110] Wallrapp A., Burkett P.R., Riesenfeld S.J., Kim S.J., Christian E., Abdulnour R.E.E., Thakore P.I., Schnell A., Lambden C., Herbst R.H. (2019). Calcitonin Gene-Related Peptide Negatively Regulates Alarmin-Driven Type 2 Innate Lymphoid Cell Responses. Immunity.

[111] Xiong L., Nutt S.L., Seillet C. (2022). Innate Lymphoid Cells: More than Just Immune Cells. Front. Immunol..

[112] Gress C., Fuchs M., Carstensen-Aurèche S., Müller M., Hohlfeld J.M. (2024). Prostaglandin D2 Receptor 2 Downstream Signaling and Modulation of Type 2 Innate Lymphoid Cells from Patients with Asthma. PLoS ONE.

[113] Tait Wojno E.D., Monticelli L.A., Tran S.V., Alenghat T., Osborne L.C., Thome J.J., Willis C., Budelsky A., Farber D.L., Artis D. (2015). The Prostaglandin D2 Receptor CRTH2 Regulates Accumulation of Group 2 Innate Lymphoid Cells in the Inflamed Lung. Mucosal Immunol..

[114] Oyesola O.O., Duque C., Huang L.C., Larson E.M., Früh S.P., Webb L.M., Peng S.A., Tait Wojno E.D. (2020). The Prostaglandin D2 Receptor CRTH2 Promotes IL-33-Induced ILC2 Accumulation in the Lung. J. Immunol..

[115] Xue L., Salimi M., Panse I., Mjösberg J.M., McKenzie A.N.J., Spits H., Klenerman P., Ogg G. (2014). Prostaglandin D2 Activates Group 2 Innate Lymphoid Cells through Chemoattractant Receptor-Homologous Molecule Expressed on TH2 Cells. J. Allergy Clin. Immunol..

[116] Petalas K., Goudakos J., Konstantinou G.N. (2023). Targeting Epithelium Dysfunction and Impaired Nasal Biofilms to Treat Immunological, Functional, and Structural Abnormalities of Chronic Rhinosinusitis. Int. J. Mol. Sci..

[117] Kato A., Kita H. (2025). The Immunology of Asthma and Chronic Rhinosinusitis. Nat. Rev. Immunol..

[118] Yang C., Guo L., Wang Y., Jiang W., Chen S., Gu Q. (2025). Correction: The Advance on Pathophysiological Mechanisms of Type 2 Chronic Rhinosinusitis with Nasal Polyposis. Front. Allergy.

[119] Shi L., Yu M., Jin Y., Chen P., Mu G., Tam S.H., Cho M., Tornetta M., Han C., Fung M.C. (2024). A Novel Monoclonal Antibody against Human Thymic Stromal Lymphopoietin for the Treatment of TSLP-Mediated Diseases. Front. Immunol..

[120] Yuan Q., Peng N., Xiao F., Shi X., Zhu B., Rui K., Tian J., Lu L. (2023). New Insights into the Function of Interleukin-25 in Disease Pathogenesis. Biomark. Res..

[121] Jia Z., Guo M., Ge X., Chen F., Lei P. (2023). IL-33/ST2 Axis: A Potential Therapeutic Target in Neurodegenerative Diseases. Biomolecules.

[122] Zhou Y., Xu Z., Liu Z. (2023). Role of IL-33-ST2 Pathway in Regulating Inflammation: Current Evidence and Future Perspectives. J. Transl. Med..

[123] Bolk K.G., Wise S.K. (2024). Biologic Therapies across Nasal Polyp Subtypes. J. Pers. Med..

[124] Zhai Z., Shao L., Lu Z., Yang Y., Wang J., Liu Z., Wang H., Zheng Y., Lu H., Song X. (2024). Characteristics of Mucin Hypersecretion in Different Inflammatory Patterns Based on Endotypes of Chronic Rhinosinusitis. Clin. Transl. Allergy.

[125] Gong X., Han Z., Fan H., Wu Y., He Y., Fu Y., Zhu T., Li H. (2023). The Interplay of Inflammation and Remodeling in the Pathogenesis of Chronic Rhinosinusitis: Current Understanding and Future Directions. Front. Immunol..

[126] Nagashima H., Mahlakõiv T., Shih H.Y., Davis F.P., Meylan F., Huang Y., Harrison O.J., Yao C., Mikami Y., Urban J.F. (2019). Neuropeptide CGRP Limits Group 2 Innate Lymphoid Cell Responses and Constrains Type 2 Inflammation. Immunity.

[127] Van Der Ploeg E.K., Carreras Mascaro A., Huylebroeck D., Hendriks R.W., Stadhouders R. (2020). Group 2 Innate Lymphoid Cells in Human Respiratory Disorders. J. Innate Immun..

[128] Gorski S.A., Hahn Y.S., Braciale T.J. (2013). Group 2 Innate Lymphoid Cell Production of IL-5 Is Regulated by NKT Cells during Influenza Virus Infection. PLoS Pathog..

[129] Lund S., Walford H., Doherty T. (2013). Type 2 Innate Lymphoid Cells in Allergic Disease. Curr. Immunol. Rev..

[130] Ogulur I., Mitamura Y., Yazici D., Pat Y., Ardicli S., Li M., D’Avino P., Beha C., Babayev H., Zhao B. (2025). Type 2 Immunity in Allergic Diseases. Cell. Mol. Immunol..

[131] Messing M., Jan-Abu S.C., McNagny K. (2020). Group 2 Innate Lymphoid Cells: Central Players in a Recurring Theme of Repair and Regeneration. Int. J. Mol. Sci..

[132] Mesuraca M., Nisticò C., Lombardo N., Piazzetta G.L., Lobello N., Chiarella E. (2022). Cellular and Biochemical Characterization of Mesenchymal Stem Cells from Killian Nasal Polyp. Int. J. Mol. Sci..

[133] Kania A.K., Kokkinou E., Pearce E., Pearce E. (2024). Metabolic Adaptations of ILC2 and Th2 Cells in Type 2 Immunity. Curr. Opin. Immunol..

[134] Corral D., Charton A., Krauss M.Z., Blanquart E., Levillain F., Lefrançais E., Sneperger T., Vahlas Z., Girard J.P., Eberl G. (2022). ILC Precursors Differentiate into Metabolically Distinct ILC1-like Cells during Mycobacterium Tuberculosis Infection. Cell Rep..

[135] Poposki J.A., Klingler A.I., Tan B.K., Soroosh P., Banie H., Lewis G., Hulse K.E., Stevens W.W., Peters A.T., Grammer L.C. (2017). Group 2 Innate Lymphoid Cells Are Elevated and Activated in Chronic Rhinosinusitis with Nasal Polyps. Immun. Inflamm. Dis..

[136] Yu Q., Song R., Ba Y., Geng J., Liu X., Liang T., Wang S., Zhao Y. (2025). Role of ILC2s as Potential Effector Cells of IL25-Mediated Type 2 Inflammation in Chronic Rhinosinusitis with Nasal Polyps in China. J. Inflamm. Res..

[137] Patel G., Pan J., Ye L., Shen X., Rosloff D., D’Souza S.S., Fung I.T.H., Celstin J., Sun W., Sankar P. (2019). Blockade of IL-4Rα Inhibits Group 2 Innate Lymphoid Cell Responses in Asthma Patients. Clin. Exp. Allergy.

[138] Kratchmarov R., Dharia T., Buchheit K. (2025). Clinical Efficacy and Mechanisms of Biologics for Chronic Rhinosinusitis with Nasal Polyps. J. Allergy Clin. Immunol..

[139] Carney A.S., Smith P.K. (2023). Current Understanding of the Role of Eosinophils in CRSwNP and Implications for Treatment with Mepolizumab and Benralizumab. Am. J. Rhinol. Allergy.

[140] Delemarre T., Holtappels G., De Ruyck N., Zhang N., Nauwynck H., Bachert C., Gevaert E. (2021). A Substantial Neutrophilic Inflammation as Regular Part of Severe Type 2 Chronic Rhinosinusitis with Nasal Polyps. J. Allergy Clin. Immunol..

[141] Kariyawasam H.H. (2020). Chronic Rhinosinusitis with Nasal Polyps: Mechanistic Insights from Targeting IL-4 and IL-13 via IL-4Rα Inhibition with Dupilumab. Expert Rev. Clin. Immunol..

[142] Le Floc’h A., Allinne J., Nagashima K., Scott G., Birchard D., Asrat S., Bai Y., Lim W.K., Martin J., Huang T. (2020). Dual Blockade of IL-4 and IL-13 with Dupilumab, an IL-4Rα Antibody, Is Required to Broadly Inhibit Type 2 Inflammation. Allergy.

[143] Lombardo N., D’Ecclesia A., Chiarella E., Pelaia C., Riccelli D., Ruzza A., Lobello N., Piazzetta G.L. (2024). Real-World Evaluation of Dupilumab in the Long-Term Management of Eosinophilic Chronic Rhinosinusitis with Nasal Polyps: A Focus on IL-4 and IL-13 Receptor Blockade. Medicina.

[144] Lombardo N., Piazzetta G.L., Lobello N., Cicala G., Patafi M., Benincasa A.T., Pelaia C., Chiarella E., Pelaia G. (2023). Real-Life Effects of Omalizumab on Chronic Rhinosinusitis with Nasal Polyposis. J. Pers. Med..

[145] Harb H., Chatila T.A. (2020). Mechanisms of Dupilumab. Clin. Exp. Allergy.

[146] Russell R.J., Boulet L.P., Brightling C.E., Pavord I.D., Porsbjerg C., Dorscheid D., Sverrild A. (2024). The Airway Epithelium: An Orchestrator of Inflammation, a Key Structural Barrier and a Therapeutic Target in Severe Asthma. Eur. Respir. J..

[147] Stanbery A.G., Smita S., von Moltke J., Tait Wojno E.D., Ziegler S.F. (2022). TSLP, IL-33, and IL-25: Not Just for Allergy and Helminth Infection. J. Allergy Clin. Immunol..

[148] Bachert C., Han J.K., Desrosiers M., Hellings P.W., Amin N., Lee S.E., Mullol J., Greos L.S., Bosso J.V., Laidlaw T.M. (2019). Efficacy and Safety of Dupilumab in Patients with Severe Chronic Rhinosinusitis with Nasal Polyps (LIBERTY NP SINUS-24 and LIBERTY NP SINUS-52): Results from Two Multicentre, Randomised, Double-Blind, Placebo-Controlled, Parallel-Group Phase 3 Trials. Lancet.

[149] Lobello N., Piazzetta G.L., Pelaia C., Preianò M., Lombardo N., Chiarella E. (2025). Patient-Derived 3D Nasal Spheroids Reveal Epithelial Changes Following Dupilumab Therapy in CRSwNP: A Preliminary Report. Tissue Barriers.

[150] Van Crombruggen K., Taveirne S., Holtappels G., Leclercq G., Bachert C. (2018). Innate Lymphoid Cells in the Upper Airways: Importance of CD117 and IL-1RI Expression. Eur. Respir. J..

[151] Li Y., Wang Z., Duan S., Wang X., Zhang Y., Bachert C., Zhang N., Wang W., Ying S., Lan F. (2025). TLR4+group 2 Innate Lymphoid Cells Contribute to Persistent Type 2 Immunity in Airway Diseases. Nat. Commun..

[152] Verma M., Michalec L., Sripada A., McKay J., Sirohi K., Verma D., Sheth D., Martin R., Dyjack N., Seibold M.A. (2021). The Molecular and Epigenetic Mechanisms of Innate Lymphoid Cell (ILC) Memory and Its Relevance for Asthma. J. Exp. Med..

[153] Palacios-García J., Porras-González C., Moreno-Luna R., Maza-Solano J., Polo-Padillo J., Muñoz-Bravo J.L., Sánchez-Gómez S. (2023). Role of Fibroblasts in Chronic Inflammatory Signalling in Chronic Rhinosinusitis with Nasal Polyps—A Systematic Review. J. Clin. Med..

[154] Chiarella E., Lombardo N., Lobello N., Piazzetta G.L., Morrone H.L., Mesuraca M., Bond H.M. (2020). Deficit in Adipose Differentiation in Mesenchymal Stem Cells Derived from Chronic Rhinosinusitis Nasal Polyps Compared to Nasal Mucosal Tissue. Int. J. Mol. Sci..

[155] Suzaki I., Maruyama Y., Kamimura S., Hirano K., Nunomura S., Izuhara K., Kobayashi H. (2024). Residual Nasal Polyp Tissue Following Dupilumab Therapy Is Associated with Periostin-Associated Fibrosis. Eur. Arch. Otorhinolaryngol..

[156] Wei Y., Ma R., Zhang J., Wu X., Yu G., Hu X., Li J., Liu Z., Ji W., Li H. (2018). Excessive Periostin Expression and Th2 Response in Patients with Nasal Polyps: Association with Asthma. J. Thorac. Dis..

[157] Balsalobre L., Pezato R., Perez-Novo C., Alves M.T.S., Santos R.P., Bachert C., Weckx L.L.M. (2013). Epithelium and Stroma from Nasal Polyp Mucosa Exhibits Inverse Expression of TGF-Β1 as Compared with Healthy Nasal Mucosa. J. Otolaryngol.-Head Neck Surg..

[158] Jansen F., Becker B., Eden J.K., Breda P.C., Hot A., Oqueka T., Betz C.S., Hoffmann A.S. (2022). Dupilumab (Dupixent^®^) Tends to Be an Effective Therapy for Uncontrolled Severe Chronic Rhinosinusitis with Nasal Polyps: Real Data of a Single-Centered, Retrospective Single-Arm Longitudinal Study from a University Hospital in Germany. Eur. Arch. Oto-Rhino-Laryngol..

[159] Poposki J.A., Klingler A.I., Stevens W.W., Suh L.A., Tan B.K., Peters A.T., Abdala-Valencia H., Grammer L.C., Welch K.C., Smith S.S. (2022). Elevation of Activated Neutrophils in Chronic Rhinosinusitis with Nasal Polyps. J. Allergy Clin. Immunol..

[160] Ohne Y., Silver J.S., Thompson-Snipes L.A., Collet M.A., Blanck J.P., Cantarel B.L., Copenhaver A.M., Humbles A.A., Liu Y.J. (2016). IL-1 Is a Critical Regulator of Group 2 Innate Lymphoid Cell Function and Plasticity. Nat. Immunol..

[161] Oka A., Klingler A.I., Kidoguchi M., Poposki J.A., Suh L.A., Bai J., Stevens W.W., Peters A.T., Grammer L.C., Welch K.C. (2025). Tezepelumab Inhibits Highly Functional Truncated Thymic Stromal Lymphopoietin in Chronic Rhinosinusitis. J. Allergy Clin. Immunol..

[162] Peters A.T., Han J.K., Heffler E., McClenahan F., Caveney S., Le T.T., Megally A., Spahn J.D., Foster A., Sherrill J.D. (2025). Thymic Stromal Lymphopoietin as a Therapeutic Target in Patients with Chronic Rhinosinusitis and Nasal Polyps. Clin. Exp. Immunol..

[163] Lupia C., Battaglia C., Pastore D., Lee Y., Piazzetta G.L., Chiarella E., Lobello N., Sireno G., Pullano A., Crimi C. (2026). Tezepelumab in Severe Asthma: Chest Computed Tomography Assessment of Airway Remodeling and Clinical Remission. Front. Pharmacol..

